# Identification and analysis of serpin-family genes by homology and synteny across the 12 sequenced Drosophilid genomes

**DOI:** 10.1186/1471-2164-10-489

**Published:** 2009-10-22

**Authors:** Matthew Garrett, Ane Fullaondo, Laurent Troxler, Gos Micklem, David Gubb

**Affiliations:** 1 Department of Genetics, Downing Street, Cambridge, CB2 3EH, UK; 2 Functional Genomics Unit, CICbioGUNE, Parque Tecnológico de Bizkaia, Ed. 801A, 48160 Derio, Spain; 3 IBMC, UPR9022 du CNRS, 15 rue Rene Descartes, F67084 Strasbourg Cedex, France

## Abstract

**Background:**

The *Drosophila melanogaster *genome contains 29 serpin genes, 12 as single transcripts and 17 within 6 gene clusters. Many of these serpins have a conserved "hinge" motif characteristic of active proteinase inhibitors. However, a substantial proportion (42%) lacks this motif and represents non-inhibitory serpin-fold proteins of unknown function. Currently, it is not known whether orthologous, inhibitory serpin genes retain the same target proteinase specificity within the Drosophilid lineage, nor whether they give rise to non-inhibitory serpin-fold proteins or other, more diverged, proteins.

**Results:**

We collated 188 orthologues to the *D. melanogaster *serpins from the other 11 Drosophilid genomes and used synteny to find further family members, raising the total to 226, or 71% of the number of orthologues expected assuming complete conservation across all 12 Drosophilid species. In general the sequence constraints on the serpin-fold itself are loose. The critical Reactive Centre Loop (RCL) sequence, including the target proteinase cleavage site, is strongly conserved in inhibitory serpins, although there are 3 exceptional sets of orthologues in which the evolutionary constraints are looser. Conversely, the RCL of non-inhibitory serpin orthologues is less conserved, with 3 exceptions that presumably bind to conserved partner molecules. We derive a consensus hinge motif, for Drosophilid inhibitory serpins, which differs somewhat from that of the vertebrate consensus. Three gene clusters appear to have originated in the *melanogaster *subgroup, *Spn28D*, *Spn77B *and *Spn88E*, each containing one inhibitory serpin orthologue that is present in all Drosophilids. In addition, the *Spn100A *transcript appears to represent a novel serpin-derived fold.

**Conclusion:**

In general, inhibitory serpins rarely change their range of proteinase targets, except by a duplication/divergence mechanism. Non-inhibitory serpins appear to derive from inhibitory serpins, but not the reverse. The conservation of different family members varied widely across the 12 sequenced Drosophilid genomes. An approach considering synteny as well as homology was important to find the largest set of orthologues.

## Background

The serpins form a family of more than 1,000 proteins found in plants, animals and viruses, but only rarely in fungi, bacteria, or archaea [1]. The majority are active **SER**ine **P**roteinase **IN**hibitors [2] with a unique cleavage mechanism. The serpin-fold consists of three β-pleated sheets with 8 or 9 short α-helical linkers. In the native state, serpins are in a metastable (stressed) configuration, which consists of a core structure with an exposed reactive centre loop (RCL) of 25-30 amino acids. The RCL presents an ideal bait to the target proteinase which cleaves between two residues known as P1 and P1'. Following cleavage, a covalent complex is formed with the target proteinase. The cut end of the RCL inserts between two β-strands within the serpin core and the molecule undergoes an extreme conformational transition to the stable (relaxed) configuration. The covalently attached proteinase is denatured by being crushed against the serpin core, which targets the complex for proteolytic degradation [3]. Such a "suicide cleavage" mechanism gives rapid turnover of both inhibitor and proteinase, with the result that a reduction in inhibitor activity gives explosive activation of proteolytic cascades. The cleavage mechanism places particular constraints on the serpin-fold, which are common to all serpin inhibitors. In particular, the suicide inhibitory mechanism requires a run of small side-chain amino acids in the proximal "hinge" region [1,4], seven residues N-terminal to the scissile bond, to allow RCL insertion into β-sheet A. This flexible hinge region is absent in the non-inhibitory serpin-fold proteins. Twelve of the 29 *D. melanogaster *serpin genes lack the critical short-side chain residues necessary for an active proteinase inhibitor [5,6].

Serpins have been extensively studied in mammals where they regulate many extracellular proteolytic cascades. The coagulation, inflammatory and complement pathways are controlled by α_1_-Antithrombin, α_1_-Antitrypsin and C1-Inhibitor, respectively [2,7,8]; while Plasminogen Activator Inhibitor-1 modulates angiogenesis, affecting both wound-healing and tumour growth [9]. There is also a related group of non-inhibitory serpin-fold proteins with diverse functions, including molecular chaperones [10,11], hormone transport [12], chromosome condensation [13], tumour suppression [14] and storage proteins [15]. In many cases, the functional requirement for the serpin-fold in the activity of these non-inhibitory molecules remains unclear.

In contrast to the mammals, insect serpins have been relatively little studied. In *Drosophila*, the Toll-mediated innate immune response is controlled by *Spn43Ac *(synonym, *necrotic*) [16,17]. The phenol-oxidase cascade is inhibited, at two different steps, by *Spn27A *[18,19] and *Spn28Dc *[20]; while tracheal melanization is regulated by *Spn77Ba *[21]. *Spn27A *also controls dorso-ventral axis formation in the embryo [22]. The alternatively spliced transcripts of the *Spn42Da *(synonym, *Spn4*) gene encode four separate RCL peptides. These protein isoforms inhibit furin-, chymotrypsin- and subtilase-like serine proteinases as well as papain-like cysteine proteinases [23-25]. Other than this, little is known about the *Drosophila *serpins, or the target proteinases that they inhibit. As there are 206 annotated chymotrypsin-fold serine proteinases in *Drosophila*, identification of target proteinases is difficult. It appears that individual serpins may inhibit proteinases in different proteolytic cascades; while particular physiological responses might be modulated by serpins targeting different steps in the same proteinase cascade.

Serpin specificity is determined by the amino acid sequence of the RCL, in particular the P1 and P1' sites, between which the peptide chain is cleaved [26]. The identity of the P1 residue is critical, with a lesser contribution of the P1' site and adjacent residues [27,28]. In general, this mechanism gives broad specificity, with each serpin inhibiting a range of related serine proteinases. This apparent "redundancy" has complicated studies of mammalian serpins, as most serpin null mutations give no discernable mutant phenotype. The lack of function of one serpin activity may be compensated by the activity of a related serpin. Furthermore, in a proteolytic cascade, the lack of inhibition of one proteinase could be compensated for by inhibition of another proteinase, by a serpin with a differing inhibitory profile. Such complex interactions are hard to analyse and are likely to have unpredictable effects on the evolutionary constraints acting on different serpin activities.

**Figure 1:**
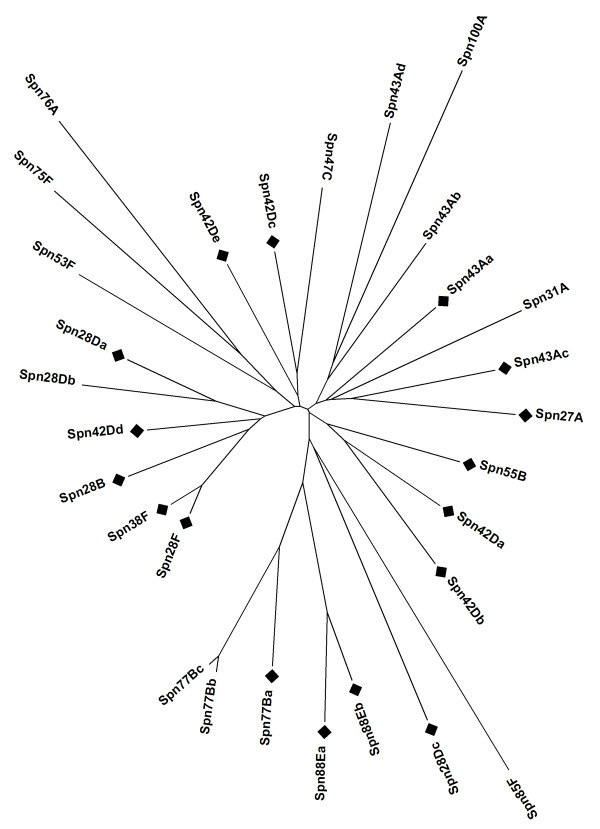
**The *D. melanogaster *serpin protein minimum evolution tree**. The serpin genes are distributed within the genome as a mixture of single transcripts and complex gene clusters. The complex loci tend to contain widely diverged transcripts, but *Spn28Da*/*Spn28Db; Spn77Ba/Spn77Bb/Spn77Ba *and *Spn88Ea/Spn88Eb *represent clusters of closely related transcripts. Inhibitory serpins are indicated with a solid black square, e.g.: *Spn43Aa*.

The vertebrate serpins have evolved through gene duplication and divergence, giving rise to a large number of transcripts encoding serpins with unique reactive centre loops and discrete physiological functions. These serpins have been divided into 9 clades of related proteins, based largely on sequence homology [1]. The number of serpins varies widely between species, with the mouse genome containing 64, compared to 35 in the human. Fitting non-vertebrate serpins within this structure is difficult, although an expanded set of 16 clades, with a number of orphan sequences, has been proposed [1]. The *D. melanogaster *serpins have been classified according to their chromosomal location, with many of them located in clusters of adjacent transcripts. This nomenclature is neutral with respect to functional similarities, but clusters of adjacent transcripts are immediately apparent. The serpins that map within polytene chromosome band 43A, for example, are identified as *Spn43Aa*, *Spn43Ab*, *Spn43Ac *and *Spn43Ad*. Of the 29 *D. melanogaster *serpin transcripts, 17 are located within 6 gene clusters. This aspect of genomic organisation has been given relatively little attention in vertebrates, but the mammalian Clade A and B serpins tend to be present in clusters and it is the relative expansion of these clusters that accounts largely for the increase of serpin transcripts in the mouse lineage [29-32]. In general, the *D. melanogaster *clusters contain widely diverged transcripts including inhibitory serpins and non-inhibitory serpin folds (Figure 1).

Although the tertiary structure of the core serpin-fold is strongly conserved, the primary sequence constraints are relatively loose [1]. The major function of the serpin core is to deliver energy stored in its metastable, stressed configuration, following cleavage of the RCL [6]. Relaxation of the serpin core distorts the active site of the proteinase and traps it in an inactive serpin/proteinase complex. This is quite different to the mechanism of action of Kunitz and Kazal-family inhibitors, which form a highly specific lock and key fit with their target proteinase. The loose sequence conservation of the serpin core presents particular problems when trying to identify the complete set of serpins in an organism. An initial homology search of the Drosophilid species available through FlyBase identifies 188 serpin genes, but even in the sibling-species, *D. simulans*, orthologues were identified for only 23 of the 29 *D. melanogaster *serpins. As the relative genomic location of putative orthologues was often conserved, we decided to use synteny as a way to check for other family members: the genes flanking annotated *D. melanogaster *serpins were used to identify the corresponding regions in the other species which were then closely examined for nearby serpin-like genes. This approach identified an additional 35 serpins. A final tBLASTx search of all 12 genomes with all 223 genes identified three further serpin genes. By analysing the sequence conservation of the serpin genes across the Drosophilidae, we address both evolutionary and structure-functional relationships within this protein super-family.

## Results and Discussion

The tBLASTx search of the Drosophilid sequence-assemblies available through FlyBase identified 188 serpin orthologues. This number represents 59% (188/319) of the orthologues expected on the assumption that the different Drosophilid species average 29 serpin genes per genome, as in *D. melanogaster*. In general, fewer orthologues were found in the out-groups, than in species close to *melanogaster *(13 serpins in the Hawaiian *D. grimshawi*, compared to 23 in *D. simulans*), Table 1. The synteny search identified all the serpin genes annotated on FlyBase, plus an additional 36 genes, Table 1 (gene identifiers are given in Additional file 1). The further tBLASTx search, using all the previously identified genes, identified three additional serpins (Table 1). These additional genes bring our coverage up to 71% of the expected orthologues (with the least coverage being 16 serpins in *D. grimshawi*, compared to 28 in *D. simulans*). Following this, we trained a hidden Markov Model against the 16 *D. grimshawi *serpin genes and searched the 12 Drosophilid genomes. This search identified 139/255 (55%) of our set of *D. melanogaster *serpins plus Drosophilid orthologues, but no additional genes. A final search for homology to the consensus RCL hinge sequence of Drosophilid inhibitory serpins,

**Table 1:** *D. melanogaster *serpins and Drosophilid orthologues

***mel***	**CG**	**P1/P1'**	**Signal** **Peptide**	**Intron No**.	***sim***	***sec***	***yak***	***ere***	***ana***	***pse***	***per***	***wil***	***moj***	***vir***	***gri***
*Spn27A*	11331	K/F	+	0	H	H	H	H	H	H	H	H	H	H	H
*Spn28B*	6717	K/K	-	1	H	H	H	H							
*Spn28Da*	31902	L/S	-	0	H	H	H	^W^H							
*Spn28Db*	33121	-	-	1	H	H	H	H							
*Spn28Dc*	7219	S/G	+	1	H	H	H	H	H	H	H	H	H	H	H
*Spn28F*	8137	Y/S	+	3	S		^*L*^*H*	^K^S	*H*^*E*^						
*Spn31A*	4804	-	-	3	H	H	H	H	H	H	H	H	H	H	H
*Spn38F*	9334	K/S	+	3											
*Spn42Da*	9453	A/S R/A T/S V/A	+/-	>3	H	S	H	H	H	H^S^	H^S^	H	H	H	H
*Spn42Db*	9454	K/G	-	2	^R^H^S^	^R^H^S^	^F^H^Y^	^R^H^S^	^R^S^S^	^R^S^S^	^R^S^S^	S	^E^S^M^	^R^S^S^	^R^S^S^
*Spn42Dc*	9455	M/M	-	2	H	S	H	H	H	H^S^	H^S^	H	S^S^	S^S^	S^S^
*Spn42Dd*	9456	R/A	+	3	S	S	^*L*^*H*^*S*^	S	S^S^	S^S^	^I^S^R^				
*Spn42De*	9460	E/S	+	3	H	H	H	H	H	H	H	S^L^			
*Spn43Aa*	12172	M/S	+	3	H	H	H	H	H	H	H	H	H	H	H
*Spn43Ab*	1865	-	+	3	H	S	H	H	H	H	H	H	H	H	H
*Spn43Ac*	1857	L/S	+	2	H	H	H	H	H	H	H	H	H^L^	H	H
*Spn43Ad*	1859	-	+	2	H	S	H	H	H	H	H		H	H	H
*Spn47C*	7722	-	-	2	H	H	H	H	H	S	S	S	S	S	S
*Spn53F*	10956	-	+	0	H	H	H	H	H						
*Spn55B*	10913	R/M	-	1	H	H	H	H	H	H	H	H	H	H	H
*Spn75F*	32203	-	-	4	H	H	H	H							
*Spn76A*	3801	-	+	1	S	S	S								
*Spn77Ba*	6680	K/A	+	>4	H	H	H	H	H	H	H	H	H^S^	H^S^	H^S^
*Spn77Bb*	6663	-	-	3	S										
*Spn77Bc*	6289	-	+	3	S	H	H								
*Spn85F*	12807	-	+	1	H	H	H	S	H	H	H	H	H	H	H
*Spn88Ea*	18525	S/A	+	2	H	H	H	H	H	H	H	H	H	H	H
*Spn88Eb*	6687	S/S	+	2	H^A^	H^A^	H^A^	H^A^							
*Spn100A*	1342	-	+	1	H	H	H	H	H	H	H	H	H	H	H

*D. melanogaster *serpins are identified by cytological location and FlyBase CG number. The putative proteinase cleavage sites (P1/P1') of inhibitory serpins are indicated in single-letter amino acid code. Putative extracellular serpins are distinguished by presence of an export signal peptide. Drosophilid species are arranged from left to right in order of increasing evolutionary separation from *D. melanogaster*: *melanogaster *sub-group (*simulans, sechellia*, *yakuba*, *erecta*), *melanogaster *group (*ananassae*), *obscura *group (*pseudoobscura*, *persimilis*), *willistoni *group (*willistoni*); *repleta *group (*mojavensis*), virilis group (*virilis*) and the Hawaiian group (*grimshawi*). Serpin orthologues were identified by homology (**H**) or synteny (**S**) to *D. melanogaster *transcripts. Three additional orthologues identified by homology, using the complete set of *Drosophilid *serpin transcripts as queries, are in *italic typeface *(***H***). The putative P1/P1' residues of inhibitory serpins are conserved within orthologous transcripts except were indicated by superscripts, left of symbol for P1 substitutions (^K^**S**), right of symbol for P1'substitutions (**H**^S^). Note that the *Spn42Da *transcript in *melanogaster *has alternatively spliced 3' and 5' exons, allowing 4 different RCL sequences to be attached to the same serpin core, either with, or without, a signal peptide. Two orthologues of *melanogaster Spn28F *were identified in ananassae.

E [EKR]G [TSG] [ET] [AGS] [YAGS] [AGS] [VAGS] [TS] (see Additional File 2), identified 75/183 (41%) of the previous inhibitory serpins, but again no novel serpin genes.

**Figure 2:**
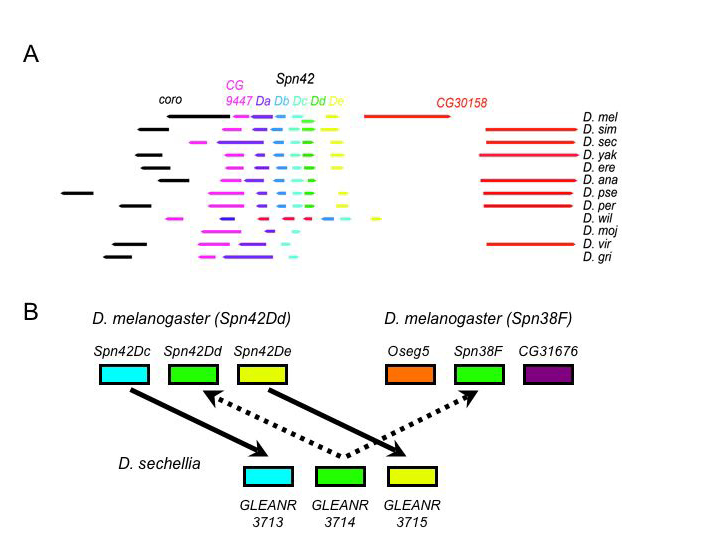
**The structure of the *Spn42D *gene cluster**. (A) The top line shows the 5 *Spn42D *serpin genes in *D. melanogaster*: *Spn42Da *(violet), *Spn42Db *(blue), *Spn42Dc *(cyan), *Spn42Dd *(green), *Spn42De *(yellow). These genes are flanked proximally by *coro *(black) and *CG9447 *(magenta) and distally by *CG30158 *(red). The orthologous genes in the other Drosophilid species are shown in successive lines. The *D. willistoni Spn42 *complex lacks a *Spn42Dd *orthologue and contains an insertion of 3 transcripts that are not syntenic with the other species, *GK20759-GK20758 *(red). Putative *Spn42Db *orthologues for *D. mojavensis, virilis *and *grimshawi *are given in Table 1, but omitted from this Figure 2 as their scaffolds are not yet located within the genome assembly. (B) The *D. sechellia *serpin *GLEANR3714 *was flagged by homology as a possible orthologue of both *Spn42Dd *and *Spn38F*. Syntenic analysis, however, shows that the adjacent transcripts from the *Spn42D *cluster, *Spn42Dc *and *Spn42De*, are plausible orthologues of the adjacent *D. sechellia *genes *GLEANR3713 *and *GLEANR3715*; while the adjacent genes to *D. melanogaster Spn38F *(*CG9333 *and *CG31676*) show no homology to the adjacent *D. sechellia *genes. By these criteria, *GLEANR3714 *is the orthologue of *Spn42Dd*, not *Spn38F*.

Three genes identified as orthologues of *Spn42Dd *and also of either *Spn28F*, or *Spn38F *were assigned as *Spn42Dd *orthologues by synteny analysis: these three genes are so closely related in *D. melanogaster *(Figure 1), that distinguishing between their orthologues by homology alone is inconclusive. The structure of the orthologous *Spn42D *gene clusters in the 12 Drosophilid species is shown in Figure 2A. The synteny search strategy is illustrated in Figure 2B. The genes flanking *D. sechellia *show homology to genes adjacent to *Spn42Dd *in *D. melanogaster*, rather than those neighbouring *Spn38F*. Thus, GLEANR 3714 is syntenic to *Spn42Dd*, not *Spn38F*. Similarly, synteny analysis indicates that *D. erecta *GLEANR 8071 and *D. pseudoobscura *GLEANR 15081 are orthologous to *Spn42Dd *not *Spn28F*.

### The distribution of serpin orthologues across the *Drosophilidae*

Orthologues of many of the inhibitory serpins were identified in all Drosophilid species, but five (out of 17) are restricted to the *melanogaster *group (*Spn28B*, *Spn28Da, Spn28Db*, *Spn28*F and *Spn88Eb*) and one (*Spn38F*) has no identified orthologues in other species. In the case of the non-inhibitory serpin-fold proteins, five have identified orthologues across the Drosophilidae (*Spn31A*, *Spn43Ab*, *Spn47C*, *Spn85F*, *Spn100A *and *Spn28B*), while the other seven show a more restricted distribution (*Spn28Db*, *Spn43Ad*, *Spn53F*, *Spn75F*, *Spn76A*, *Spn77Bb *and *Spn77B*c) (Table 1).

### Using synteny to find additional family members

Within multi-gene families, identifying the true orthologues of a particular gene becomes increasingly difficult in distantly related species. Here we have used conservation of gene order in the chromosome, both as a means to search for additional family members, and also as a way of assigning putative orthology in otherwise ambiguous cases. This approach is based on the finding that the order of the majority of the genes along the chromosome arms is conserved among the Drosophilid species [33-36]. This allows neighbouring transcripts to be used to identify putative serpin genes which may have been missed by homology-dependent searches. Using this approach, we identified 223 serpin genes in the Drosophilid lineage with conserved synteny to *D. melanogaster*, compared to 188 genes identified by sequence homology alone. A tBLASTx homology search using this expanded set of 223 genes as queries, identified 3 additional orthologues, bringing the total number of identified serpin genes to 226. The identity of candidate syntenic genes was confirmed by aligning their putative RCL sequences with that of the corresponding set of orthologues (Table 1 and see Additional file 2). By this criterion the synteny approach made no false assignments. In addition to increased identification of serpin transcripts, the method allows the assignment of putative orthology when considering closely-related genes.

There were three cases in which the tBLASTx and synteny searches failed to identify orthologues in a single species (*D. sechellia Spn43Ab, D. willistoni Spn43Ad *and *D. erecta Spn85F*). Examination of the sequence alignment of the syntenic genomic regions by eye identified the missing orthologues for *D. sechellia Spn43Ab *and *D. erecta Spn85F*, but the *D. willistoni Spn43Ad *transcript is deleted from the orthologous *Spn43A *gene complex.

If each of the Drosophilid species contained 29 serpin genes per genome, as in *D. melanogaster*, the synteny approach identifies 71% of the expected serpin genes. It is notable, however, that even in the sibling-species, *D. simulans*, only 23 orthologues were identified by sequence homology, with 5 more identified by synteny, Table 1. The number of identified serpin genes decreases uniformly with increasing evolutionary separation from *D. melanogaster*. Only 13 serpins are identified by sequence homology (and 16 by synteny) in *D. grimshawi*. While the exact number of serpins may vary among fruit-flies, it seems unlikely that this number should correlate with evolutionary separation from *D. melanogaster*. This progressive decrease implies that many of the "missing" serpin functions may be represented by transpositions of serpin genes to novel chromosome sites, followed by sequence divergence. Investigations aimed at identifying further serpin family members are described below.

### Conservation of RCL sequences in serpin genes

Many of the RCL sequences are strongly conserved across the *Drosophilid *lineage, over 40 million years. This is particularly the case with putative inhibitory serpins, Additional file 2. The general RCL consensus hinge region sequence (P17-P9) for inhibitory serpins is E [EKR]G [TS].(AGS)_4 _[1], being the 17-9th residues N-terminal to the P1/P1'proteinase cleavage site. In the *Drosophilid *lineage the hinge consensus includes the P8 residue, which is constrained to [TS] and the P13 residue is predominantly [ET]. The presence, or absence, of the hinge-region consensus E [EKAQR]G [TSG] [ET] [AGS] [YAGS] [AGS] [VAGS] [TS] separates the Drosophilid serpins unambiguously into two classes (see Additional file 2), with the exception of *Spn100A*, see below.

**Figure 3:**
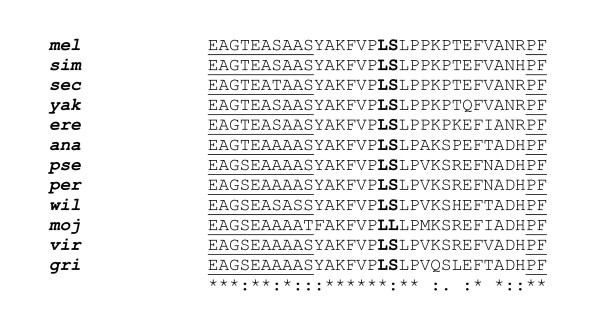
**Multiple sequence alignment of the RCL of *Spn43Ac *orthologues**. Alignment annotation: * Identical residues: closely similar residues, broadly similar residues. The conserved residues in the flexible hinge region and the PF residues of the "shutter region" are underlined. The putative proteinase cleavage site (P1/P1') residues are marked in **bold **typeface.

The RCL sequences of the majority of inhibitory serpins are strongly conserved among species, with almost complete identity of the putative P1/P1' sites (*Spn27A*, *Spn28B*, *Spn28Da*, *Spn28Dc*, *Spn42Dc*, *Spn43Aa*, *Spn43Ac*, *Spn55B, Spn77Ba, Spn88Ea *and *Spn100A*), Table 1, Figure 3 and Additional file 2. There are three exceptions to this general rule, where the evolutionary constraints appear to be looser: *Spn28F, Spn42Db *and *Spn42Dd*. The orthologues of these three serpins show divergent RCL sequences, with variable P1/P1'sites affecting specificity for putative target proteinases. That these RCL sequences were able to diverge implies that their corresponding ancestral serpins were, at least partially, redundant genetic functions.

In general, the substrate specificity of many serpins is broad and loss of the activity of one serpin may be compensated by a related activity. Where this occurs, the constraints on RCL sequence divergence will be less stringent than for strictly non-redundant serpin functions. In addition, the loss of inhibition of a particular proteinase could be compensated by inhibition of a different proteinase within the same cascade, by a serpin with a different inhibitory spectrum. By the criterion that they have conserved P1/P1'sites, the majority of inhibitory serpins in the Drosophilid genomes are non-redundant. In *D. melanogaster*, visible or lethal phenotypes are associated with lack of function mutations (*Spn27A *[18,19,22], *Spn28Dc *[20]*Spn42Da *[23-25,37,38], *Spn43Ac *[16,17], *Spn77Ba *[21]). The Japanese collection of RNAi knockdown strains includes eight of the seventeen inhibitory serpins (Table 1). Of these, 5 give a lethal phenotype (*Spn27A, Spn38F, Spn43Aa*, *Spn43Ac *and *Spn55B*) and three (*Spn28F, Spn42De *and *Spn55B*) are viable . These data sets are incomplete, but again indicate that many of the inhibitory serpins represent non-redundant genetic functions. There is no evidence for a general expansion of transcripts within complex loci as occurred within the vertebrate A and B serpin clades [29,30,32,39].

In contrast, the RCLs of non-inhibitory serpin-fold proteins (*Spn28Db*, *Spn31A*, *Spn43Ab*, *Spn43Ad*, *Spn47C*, *Spn53F*, *Spn76A*, *Spn77Bb*, *Spn77Bc*, *Spn85F *and *Spn100A*) are weakly conserved, implying relaxed sequence constraints, Additional file 2. There are three exceptions, in which the RCL is extremely conserved (*Spn31A*, *Spn43Ab *and *Spn85F)*. The RCLs of these three serpin-folds presumably bind to strongly-conserved partner molecules. Four (out of 12) of the non-inhibitory *melanogaster *serpin-folds have orthologues restricted to the *melanogaster *group.

The serpin core structure, of 3 β-pleated sheets and 8 or 9 α-helical linkers would be expected to be relatively unconstrained within the Drosophilid lineage. We find that the alignment scores between *D. melanogaster *and *D. grimshawi*, separated by about 40 Myr, are higher for the RCL sequences of inhibitory serpins than for their serpin cores (Table 2). In the non-inhibitory serpin-fold proteins, the alignment scores of the core and RCL regions are similar, with three exceptions where the RCL sequence is strongly conserved (*Spn31A, Spn43Ab *and *Spn85F*) and one where both core and RCL are moderately conserved (*Spn100A*) (Table 2).

**Table 2:** Sequence conservation in serpin core and RCL between *D. melanogaster *and *D. grimshawi*

**Serpin**	**P1/P1'** **mel**	**P1/P1'** **gri**	**Alignment Score** **%** **Serpin core**	**Alignment Score** **%** **RCL**
*Spn27A*	K/F	K/F	70	87
*Spn28Dc*	S/G	S/G	45	76
*Spn31A*	-	-	67	80
*Spn42Da*	A/S	A/S	66	69
*Spn42Db*	K/G	R/S	46	49
*Spn42Dc*	M/M	M/S	52	75
*Spn43Aa*	M/S	M/S	80	95
*Spn43Ab*	-	-	77	90
*Spn43Ac*	L/S	L/S	41	72
*Spn43Ad*	-	-	53	46
*Spn47C*	-	-	41	44
*Spn55B*	R/M	R/M	76	94
*Spn77Ba*	K/A	K/S	58	72
*Spn85F*	-	-	68	96
*Spn88Ea*	S/A	S/A	55	84
*Spn100A*	-	-	62	67

The putative proteinase cleavage sites (P1/P1') of inhibitory serpins rarely change between the *D. melanogaster *and *D. grimshawi *orthologues and the RCL sequence is more strongly conserved than the serpin core. The RCL of non-inhibitory serpin-folds tends to be weakly conserved, with three exceptions, *Spn31A*, *Spn43Ab *and *Spn85F*. The putative RCL of the serpin-related fold, *Spn100A*, is conserved slightly more strongly than the remainder of the protein.

### Structure of serpin gene clusters

The *Spn42D *gene cluster contains widely diverged transcripts in *melanogaster *(Figure 1) corresponding to ancient duplication events that are conserved across the most of the *Drosophilidae*. Within the *Spn42D *complex, the *Spn42Db *and *Spn42Dd *genes show particularly labile RCL sequences, however, the overall structure of the complex locus is maintained. The structure of the alternatively-spliced *Spn42Da *transcription unit is maintained within the 5 species of the *melanogaster *sub-group, but the RCL sequences diverge increasingly in the 7 outgroup species [38]. Similarly, the structure of the *Spn43A *complex is conserved, apart from the loss of the *Spn43Ab *gene in *D. sechellia *and the *Spn43Ad *gene in *D. willistoni*. In general, there is little evidence of variation of number of genes within complex loci, such as occurs in the vertebrate Clade A and Clade B lineages [39,30-32].

The *Spn77B *and *Spn88E *gene clusters, on the other hand, contain transcripts with closely related sequences in *melanogaster *(Figure 1). The duplication/divergence events which gave rise to these clusters apparently occurred within the last 5 Myr in the *melanogaster *subgroup [40,41]. The ancestral gene for the *Spn77B *cluster appears to be the inhibitory serpin *Spn77Ba*, which has orthologues in all the *Drosophilid *species. The *Spn77Bb *gene is unique to *D. melanogaster*, while the closely homologous *Spn77Bc *gene is found in *D. melanogaster*, *D. simulans *and *D. sechellia*. The *Spn77Bb *and *Spn77Bc *genes, therefore, appear to have arisen from recent duplication events; they are both non-inhibitory serpin-fold proteins, one putatively cell-autonomous (lacking an export signal) and the other secreted (carrying an export signal). Similarly, with the inhibitory *Spn88Ea *and *Spn88Eb *serpins, the ancestral gene would be *Spn88Ea*, with an RCL loop that is extremely conserved across the 12 species. The *Spn88Eb *gene appears to have arisen as a tandem duplication within the *melanogaster *sub-group and retains the S/A P1/P1' sites of *Spn88Ea*, except in *melanogaster*, where it is S/S. We have here an example of duplication of a serpin gene, divergence of the RCL sequence and, finally, modification of the putative proteinase cleavage site in *melanogaster *itself.

Similarly, the inhibitory *Spn28Da *and non-inhibitory *Spn28Db *serpins, are adjacent, closely related genes (Figure 1). They appear to represent duplications in the *melanogaster *group. The most likely ancestral sequence is the inhibitory *Spn28B*, which has a single intron, like *Spn28Db*. The *Spn28Da *duplication event was associated with loss of an intron and divergence of the inhibitory RCL sequence. The *Spn28Db *duplication event retained the single intron, but lost the conserved flexible hinge-residues characteristic of inhibitory RCL sequences. In general, it should be more frequent for non-inhibitory serpin-fold to evolve from an active inhibitor than the reverse; a single substitution within the conserved hinge-region motif can inactivate the inhibitory mechanism [4]; while a series of changes in a non-inhibitory serpin-fold would be required to generate the hinge-region consensus. We did not identify any sets of orthologues that contained both inhibitory and non-inhibitory serpins, which ought to have been identified by the synteny approach. The absence of such mixed sets of inhibitory and non-inhibitory orthologues again implies that the evolution of serpin function is constrained, in the absence of duplication/divergence events.

### Sporadic loss and gain of individual genes

The *D. melanogaster *minimum evolution tree (Figure 1) indicates that the *Spn28F*, *Spn38F, Spn28B*, *Spn42D, Spn28Db *and *Spn28Da *genes are related. Within this group, *Spn28F *and *Spn38F *are closely related and, like *Spn42Dd*, contain 3 introns. The transcripts of the *Spn28 *cluster, on the other hand, contain one, or no, introns and, on this basis, represent a separate sub-group. The identified orthologues of all six of these genes are restricted to the *melanogaster *species sub-group, with the exception of *Spn42Dd*, which also has identified orthologues in *ananassae *and the *pseudobscura *sub-group. The most parsimonious interpretation of the relationship of the *Spn28F *and *Spn38F *genes is that they represent recent duplication/divergence events within the *melanogaster *sub-group lineage. Similarly, we identified two orthologues for *Spn28F *in *D. ananassae*, which represent a presumptive duplication in this species, and failed to identify a *Spn28F *orthologue in *D. sechellia*, indicating a presumptive loss. As mentioned above, the putative P1/P1' sites of the *Spn28F *orthologues are variable within the *melanogaster *group implying a degree of functional redundancy; which is consistent with the loss of this orthologues in *D. sechellia*.

Apart from these instances there is little evidence of loss and gain of serpin genes. As discussed in the previous section, the *Spn77B *gene cluster in *melanogaster*, represents a set of recent duplication events, the *Spn88Eb *gene corresponds to a recent duplication in the *melanogaster *sub-group and the *Spn43Ad *gene is missing in *D. willistoni*. In general, loss and gain of serpin genes is rare in the Drosophilid lineage.

That said, we might have failed to detect additional transposition/divergence events in the more distant species to *D. melanogaster*. If such events had occurred, the loose sequence constraints on the serpin core would allow transposed genes to diverge rapidly, so that they would be difficult to identify by homology. Furthermore, single amino-acid substitutions in the RCL of an inhibitory serpin can completely alter its substrate specificity [42]. The functions of the inhibitory serpins with orthologues restricted to the *melanogaster *sub-group could be replaced in the out-groups by serpins carrying similar P1/P1' residues in their RCLs attached to very distantly related serpin cores. Thus the *melanogaster *outgroups may well have additional serpin genes that we fail to identify by either homology or synteny.

A particular case of divergence/duplication of serpin inhibitory function can occur within the alternatively-spliced RCL-encoding exons of *Spn42Da*. Alternatively-spliced serpins were first identified in *Bombyx mori *[43], although the genetic mechanism was only later described in *Manduca sexta *[44,45]. Similar alternative-exon cassettes have been described in *Anopheles *[46] and Caenorhabditis [47], although to date no mammalian examples are known. In the *Drosophilid *lineage, the furin-inhibitory RCL (exon 5) is maintained in all species as well as exons 6-8 in the *melanogaster *sub-group, but the corresponding exons duplicate and diverge in the 7 outgroup species [38]. It might be expected that some of the inhibitory serpin orthologues that we failed to identify in the outgroup species were complemented by inhibitory functions on the alternative-exon cassettes of the *Spn42Da *orthologues. This appears not to be the case by the criterion that the putative P1/P1'sites of these *melanogaster *serpins (i.e: K/K, L/S, Y/S, K/S, R/A, E/S and S/S; corresponding to *Spn28B*, *Spn28Da*, *Spn28Db*, *Spn38F, Spn42Dd, Spn42De *and *Spn88Eb*) see Table 1, do not correspond to the P1/P1'sites on the novel alternative-exon cassettes in the outgroup species (i.e: *D. ana *S/R, C/S, L/S; *D. per *C/S, S/R; *D. pse *C/S, S/R; *D. wil *R/S, S/A, S/Y, C/S, R/I; *D. moj *L/T, E/M; *D. vir *R/T, E/M, D/S and *D. gri *G/M, E/M, M/L, D/M, R/A, L/M), information extracted from Figures 2, 3, 4 [38].

### Conservation of N-terminal and serpin core sequences of Spn43Ac and Spn100A

The serpins are generally between 350 and 400 amino-acids in length, with very few having either N- or C-terminal extensions to the core serpin domain. One of the few human serpins with an N-terminal peptide, Heparin cofactor II, shows reduced substrate specificity after N-terminal cleavage [48]. Similarly, the *Drosophila melanogaster Spn43Ac *(*Nec*) serpin has a long N-terminal extension, which is cleaved on immune challenge [49]. In the case of *Spn43Ac*, both the full length protein and the cleaved core serpin are active proteinase inhibitors *in vitro*, but the core serpin shows increased substrate specificity for porcine pancreatic elastase (PPE) [49]. Alignment of the *Spn43Ac *orthologues shows that the N-terminal extension is present throughout the *Drosophilidae *(see Additional file 3). This peptide varies in length between 69 amino acids (*D. sechellia*) and 145 amino acids in (*D. grimshawi*) with poor sequence conservation, other than the putative R/P scissile bond (see Additional file 3). A long, unstructured N-terminal extension appears to be critical for the function of the *Spn43Ac *orthologues, but its precise length and sequence are weakly constrained. Note the poor sequence conservation within *Spn43Ac *serpin core sequence, with the exception of the exposed RCL.

The *Spn100A *transcript encodes a putative 653 amino acid protein, which is outside the normal serpin size-range. The sequence shows two blocks of serpin homology: an N-terminal stretch of 44 amino acids and a C-terminal stretch of 235 amino acids, separated by an insertion of 294 amino-acids (Pfam alignment) (see Additional file 4). This structure is supported by comparison of genomic and cDNA sequences, which show a single intron of 87 bp in *D. melanogaster*. The novel internal segment of 294 amino acids can not, therefore, correspond to read-through of a nested transcript. The C-terminal of the Spn100 protein includes a putative RCL, including some of the flexible hinge region residues characteristic of inhibitory serpins. The alignment of the N- and C-terminal serpin domains is conserved in all 11 Drosophilid orthologues, and the amino-acid sequence conservation is similar across the whole length of the protein. The implication is that the *Spn100A *gene encodes a novel, serpin-derived, protein fold. Novel protein folds arise extremely infrequently and a mechanism involving chimeric transcripts formed from segments of pre-existing peptide-coding sequence has been proposed [50]. Database searches identify *Spn100A *orthologues in mosquitoes and other insects, but not in other organisms.

### Comparison of Drosophilid and vertebrate serpins

Vertebrate genomes contain a large, but variable number, of serpins and proteinases. The human complement of 35 serpins and 176 chymotrypsin-fold serine proteinases is increased to 64 and 227 in the mouse, with many of the differences being in genes involved in reproduction and immunity [51]. Against this background, the *D. melanogaster *genome contains 29 serpins and 211 chymotrypsin-fold serine proteinases, with *Spn42Da *encoding 11 isoforms, with or without a signal peptide. A large proportion of these serpins have immune-related functions, or are expressed in the reproductive track. Comparison of human and mouse genomes shows that the serpins of nine of the vertebrate clades show conserved numbers and synteny, with the variations being restricted to clades A and B. In particular, the mouse clade A serpins have expanded, with human α_1_-antitrypsin being represented by a cluster of 5 genes in the mouse, and human α_1_-Antitrypsin being represented by a cluster of 14 genes. Similarly, 4 of the human clade B serpins are represented by clusters of 3 to 7 genes in the mouse [29,31,32]. A striking feature of this expansion in the mouse lineage, is that many of the RCL sequences are unique [29], novel sets of proteinases are being targeted. We find no evidence of a similar massive amplification of serpin genes in the Drosophilid lineage, although the *melanogaster Spn77B *and *Spn88E *clusters appear to represent recent tandem duplications. The one example of extensive duplication/divergence of serpin inhibitory functions that has been identified in the Drosophilid lineage is within the novel alternative-exon cassettes of the *Spn42Da *orthologues [38]. As in the mouse clade A and B expansions, novel sets of proteinases are being targeted by the diverged alternative-exon cassettes (see above).

An essential component of the serpin suicide-inhibition mechanism, is that the cut RCL sequence inserts between two strands of β-sheetA, as the metastable serpin structure relaxes. As a consequence, it is possible for the RCL of one serpin to insert between β-sheetA of another in the absence of enzymatic cleavage, to form an inert, stable polymer [52,53]. Under normal physiological conditions, this tendency is low, but it represents a constraint on serpin evolution and mutations which alter the stability of the serpin core can favour polymer formation. Such mutations underlie a set of human genetic diseases, including the Z-allele α_1_-antitrypsin serpinopathy [54] and conformational dementias [55]. Similar constraints on the stability of the serpin core have been demonstrated in *Drosophila*, with the isolation of polymerogenic mutations of *Spn43Ac *(Necrotic) showing homologous amino acid substitutions to Z-allele α_1_-antitrypsin and other pathogenic serpin variants [56,57].

## Conclusion

We have developed a synteny search algorithm which is a powerful tool for identifying orthologous members of multi-gene families within related species. In the Drosophilid lineage, the majority of inhibitory serpin orthologues show a striking conservation of the critical the P1/P1' proteinase cleavage sites, indicating that these inhibitors do not readily change specificity for their target proteinases. Despite this, the distribution of orthologues is quite patchy: many *D. melanogaster *serpins have no identified orthologues in the more distant species. The balance between inhibitory serpins and the target proteinases that they control forms a strong evolutionary constraint. The majority of Drosophilid inhibitory serpin orthologues appear to represent non-redundant genetic functions, with strong constraints on the P1/P1'sites. Three complex loci in *D. melanogaster *appear to represent recent duplication/divergence events where the sequence constraints have been relaxed. Each complex contains a putative ancestral inhibitory serpin, with orthologues across the whole Drosophilid lineage, plus one or two genes restricted to the *melanogaster *group. Taken together our analyses imply that evolution within the Drosophilid serpin super-family has occurred predominantly by a gene duplication/divergence mechanism.

## Methods

To assemble a curated set of serpin sequences within the *Drosophilidae*, we first compiled the orthologues from a tBLASTx search of the 12-species genes extracted from FlyBase . Additional serpins were identified using a synteny based approach, which relies on the conservation of the order of chromosomal transcripts between related species. The synteny search code is given in Additional file 5. Genes adjacent to serpin genes in *Drosophila melanogaster *were used to identify the corresponding region within the other *Drosophilid *genome sequence assemblies. Where the two adjacent genes on either side of a *Drosophila melanogaster *serpin could be uniquely resolved to orthologues in other species, the intervening sequence was examined for putative serpin genes. Where the adjacent gene orthologues could not be resolved in a given species, the process was repeated with more distant genes, up to a maximum of 3 genes from the original *D. melanogaster *serpin. Any putative matches were compared to the original serpin protein by tBLASTx alignment [58]. High-scoring matches were considered to be likely orthologues on the basis of both synteny and sequence conservation. A final tBLASTx search was made using all the Drosophilid serpin genes identified in the previous searches as queries against all 12 *Drosophilid *genomes. The sets of serpin amino-acid sequences were aligned using CLUSTALW. Minimum evolution bootstraps were built using MEGA4 [59] for each serpin within each *Drosophilid *species.

## Abbreviations

Serpin (Spn): Serine proteinase inhibitor; RCL: Reactive centre loop; P1/P1': proteinase cleavage site; Myr: million years.

## Authors' contributions

MG wrote the synteny search code and extracted the orthologous genes from the genomic sequence databases. AF collated and analysed the sequence data. DG and AF wrote the manuscript. LT identified and corrected several alignment errors and incomplete gene sequences. GM participated in the design of search strategies and critically reviewed the manuscript.

## Supplementary Material

Additional file 1 **Gene identifiers of the *D. melanogaster *serpins and their Drosophilid orthologues**. Table giving the CG numbers for the D. melanogaster serpins and the GLEANR numbers of their identified orthologues in other Drosophilid species. Click here for file

Additional file 2 **Multiple alignment of Drosophilid serpin RCL sequences**. Table showing the alignment of the critical RCL region of orthologous Drosophilid serpins. The conserved flexible hinge region of the putative inhibitory serpins are indicated in red, with the putative scissile bond, P1/P1'site, indicated in green. Click here for file

Additional file 3 **Multiple sequence alignment of Drosophilid *Spn43Ac *orthologues**. Alignment of the *Spn43Ac *orthologues indicate that a long N-terminal extension is conserved across the Drosophilid species, although sequence conservation is poor, and the length varies between 69 and 145 amino acids. Click here for file

Additional file 4 **Multiple sequence alignment of Drosophilid *Spn100A *orthologues**. The alignment of *Spn100A *orthologues indicates that this gene is a serpin-related fold showing two blocks of serpin homology separated by an insertion of 294 amino-acids. Click here for file

Additional file 5 **Synteny search code**. Code used to search for orthologous genes by synteny of adjacent transcripts. Click here for file
