# Supplementary material for: Identification and analysis of serpin-family genes by homology and synteny across the 12 sequenced Drosophilid genomes
Source: BMC Genomics. 2009 Oct 22;10:489. doi: 10.1186/1471-2164-10-489 (PMC2770083; doi:10.1186/1471-2164-10-489)
Supplement: Additional file 1 — Gene identifiers of the D. melanogaster serpins and their Drosophilid orthologues. Table giving the CG numbers for the D. melanogaster serpins and the GLEANR numbers of their identified orthologues in other Drosophilid species. [file 1471-2164-10-489-S1.PDF]

D. mel Cytol.	*D. mel* CG	*sim*	*sec*	*yak*	*ere*	*ana*	*pse*	*per*	*wil*	*moj*	*vir*	*gri*
Spn27A	11331	6302	1481	2208	8406	15219	7349	8165	19703	931	677	13479
Spn28B	6717	7211	1762	1472	10405							
Spn28Da	31902	6208	1384	19274	16812							
Spn28Db	33121	6207	1383	2113	8310							
Spn28Dc	7219	6200	1377	1135	8303	16100	1787	20654	15403	1765	1813	11349
Spn28F	8137	6177		1112	8282	21984<br>16627						
Spn31A	4804	7391	1944	2680	10043	16506	2233	21101	18779	2390	2502	10974
Spn38F	9334											
Spn42Da	9453	10254	3650	809	10682	11794	14922	11088	3537	4717	329	4163
Spn42Db	9454	10253	3648	808	10681	11793	14921	11087	3533	10088	8709	2579
Spn42Dc	9455	10416	3713	2857	8070	13160	15080	11268	3651	4629	213	6122
Spn42Dd	9456	10417	3714	*2858*	8071	13161	15081	23015				
Spn42De	9460	10419	3715	2859	8072	13162	15082	11269	3652			
Spn43Aa	12172	10445	3742	2885	8099	12716	15110	11299	4888	4418	15447	5673
Spn43Ab	1865	10233	3625	791	10661	12251	14907	11072	4638	4937	15254	4634
Spn43Ac	1857	10231	3622	789	10659	12249	14905	11070	4637	4935	15252	4632
Spn43Ad	1859	10232	3623	790	10660	12250	14906	11071		4936	15253	4633
Spn47C	7722	9934	3299	13303	7409	12447	16411	2309	3449	4671	275	4806
Spn53F	10956	9485	2825	14352	6961	11498						
Spn55B	10913	11307	4596	12212	6622	11729	14523	10659	5346	4356	7768	4518
Spn75F	32203	12368	15644	6673	13689							
Spn76A	3801	12376	13628	6196								
Spn77Ba	6680	14870	5059	3522	16113	10285	12901	7505	17292	13312	11539	14584
Spn77Bb	6663	14873										
Spn77Bc	6289	14871	5060	3523								
Spn85F	12807	2474	6874	9582	17291	19112	4400	9813	11417	7462	9634	683
Spn88Ea	18525	4158	8740	9950	562	18423	3417	3776	14296	8594	10154	2976
Spn88Eb	6687	4157	8739	9949	561							
Spn100A	1342	5295	13268	10783	11836	7460	4684	13978	14468	7360	9531	14103

D. melanogaster serpins are identified by cytological location and FlyBase CG number. Serpin orthologs in the Drosophilid species (*simulans, sechelia*, *yakuba*, *erecta*, *ananassae*, *pseudoobscura*, *persimilis*, *willistoni*, *mojavensis*, *virilis* and *grimshawi*) are identified by their GLEANR numbers.
