# Supplementary material for: Identification and analysis of serpin-family genes by homology and synteny across the 12 sequenced Drosophilid genomes
Source: BMC Genomics. 2009 Oct 22;10:489. doi: 10.1186/1471-2164-10-489 (PMC2770083; doi:10.1186/1471-2164-10-489)
Supplement: Additional file 2 — Multiple alignment of Drosophilid serpin RCL sequences. Table showing the alignment of the critical RCL region of orthologous Drosophilid serpins. The conserved flexible hinge region of the putative inhibitory serpins are indicated in red, with the putative scissile bond, P1/P1'site, indicated in green. [file 1471-2164-10-489-S2.PDF]

Serpin	Multiple Alignments of R C L Sequences

```
                           P17----------P8---------P1/P1'---------------P14'PF

Spn27A      mel            EKGTEAYAATVVEIENKFGGSTAIEEFNVNRPF
(CG11331)   sim            EKGTEAYAATVVEIENKFGGSTAIEEFNVNRPF
            sec            EKGTEAYAATVVEIENKFGGSTAIEEFNVNRPF
            yak            EKGTEAYAATVVEIENKFGGSTTIEEFNVNRPF
            ere            EKGTEAYAATVVEIENKFGGSTLIEEFNVNRPF
            ana            EKGTEAYAATVVEIENKFGGSTIIEEFNVNRPF
            pse            EKGTEAYAATVVEIENKFGGSTTIEEFNVNRPF
            per            EKGTEAYAATVVEIENKFGGSTTIEEFNVNRPF
            wil            EKGTEAYAATVVEIENKFGGSIVIEEFNVNRPF
            moj            EQGTEAYAATVVEIENKFGGSQTIVEFNVNRPF
            vir            EQGTEAYAATVVEIENKFGSSQTIVEFNVNRPF
            gri            EQGTEAYAATVVEIINKFGGPTKIEEFNVNRPF
                           *:************ ****..  * ********

Spn28B      mel            EKGGEASAATGVLTRRKKSIDNLIQPPMEFIADHPF
(CG6717)    sim            EKGGEASAATGILTRRKKSIDNLRLPPMEFIVDHPF
            sec            EKGGEASAATGVLTRRKKSIDNLRLPPMEFIVDHPF
            yak            ERGTKASAATGVLIRRKKSIDNLKPPPMEFVADHPF
            ere            ERGAKASAATGVLIRPKKSIDNMKLPPMEFIADHPF
                           *:* :******:* * ******:  *****:.****

Spn28Da     mel            EEGGSAGSASASPIRGLSDYATSVVTFTVNSPF
(CG31902)   sim            EEGGSAGSASANPIRGLSDYAASLVTFTVNSPF
            sec            EEGGSAGSASANPIQGLSDYAASVVTFTVNSPF
            yak            EEGASAGSASASLFRGLSDYPASVASFTVNSPF
            ere            EEGASAGSASASLLRGWSDYPANVASVRVDRPF
                           ***.*******. ::* ***.:.:.:. *: **

Spn28Db     mel            HNKKVYVRMMSHVGRFRIADHSYGQIIEMPF
(CG33121)   sim            HNRKAYVRMMSHVGRFRIADHSYGQIIELPF
            sec            HNREAYVRMMSHVGRFRIADHSYGQIIELPF
            yak            HNKKVYVRMMSHVGRFRMADQSYGQIIELPF
            ere            HNKAVHVRMMSQVGRFRMAKQSYGQIIELPF
                           **: :: *****:******::*******:**

Spn28Dc     mel            EQGTEAAASSVTYLKKSGPDVLFRGDTPF
(CG7219)    sim            EQGTEAAASSVTYLKKSGPDVLFRGDTPF
            sec            EQGTEAAASSVTYLKKSGPDVLFRGDTPF
            yak            EQGTEAAASSVTYLKKSGPDVVFRGDTPF
            ere            EQGTEAAASSVTYLKKSGPDVVFRGDTPF
            ana            EQGTEAAAATVTYLKKSGPDVLFRGDTPF
            pse            EQGTEAAAATVTYVKKSGPDVLFRGDTPF
            per            EQGTEAAAATVTYVKKSGPDVLFRGDTPF
            wil            EQGTEAAAATIAYLKKSGPDILFRVDTPF
            moj            EQGTEAAAATAAILKKSGPEVLFRAETPF
            vir            EQGTEAAAASAAILKKSGPEVVFRAETPF
            gri            EQGTEAAAASAAVLKKSGPDVVFRAETPF
                           ********:: : :*****:::** :***

Spn28F      mel            EEGAEAAAATALLFVRYSMP-MPSSQMVFNADHPF
(CG8137)    sim            EEGAEAAAATALLTVRYSKP-RPSSQMVFNADHPF
            yak            EEGAEAAAATKIVVYPLS---AHPIWMDFNVDHPF
            ere            EEGAEAAAITKIDMVFKSASRPQPI--DFIADHPF
            ana (16627)    EEGAEAAAVTAVVAVHICANCATPSRMSFIANHPF
            ana (21984)    EEGAEASAASAVSIVFYE--------MDFHADHPF
                           ******:* : :                * .:***
```

```
Spn31A      mel   ESGSGSGPELPKNATEYKPIVISNSSRQKFFRADHPF
(CG4804)    sim   ESGSGP--ELPKNATEYKPIVISNSSRQKFFRADHPF
            sec   ESGSGP--ELPKNATEYKPIVISNSSRQKFFRADHPF
            yak   ESGSGP--ELPKNATEYKPIVISNSSRQKFFRADHPF
            ere   ESGSGP--ELLKNATEYKPIVISNSSRQKFFRADHPF
            ana   ESGSGS--EHPRVAAEYKPIVISNSSRQKFFRADHPF
            pse   ESGSGS--EPPRAAAEYKPIVISNSSRQKFFRADHPF
            per   ESGSGS--EPPRAAAEYKPIVISNSSRQKFFRADHPF
            wil   ESGSGS--ESPQPAAAYKPIVISNSSRQKFFRADHPF
            moj   ESGSGS--ELPPPATGYKPIVISNSSRQKFFRADHPF
            vir   ESGSGS--ELPQPAAGYKPIVISNSSRQKFFRADHPF
            gri   ESGSGS--ESPQPAAGYKPIVISNSSRQKFFRADHPF
                  *****.  *    *: *********************:

Spn38F      mel   EEGSEAAAATAVVFRYKSIRSP-PMDFNVNHPF
(CG9334)

Spn42Da     mel   EEGTEAAAATGMAVRRKRAIMSPEEPIEFFADHPF
(CG9453)    sim   EEGTEAAAATGMAVRRKRAIMSLEEPIEFLADHPF
            sec   EEGTEAAAATGMAVRRKRAIMSLEEPIEFLADHPF
            yak   EEGTEAAAATGMVVRRKRAIVSLEEPLEFLADHPF
            ere   EEGTEAAAATGMVARTKRAIYSLEEPIDFFADHPF
            ana   EEGTEAAAATGFRIMPVAGFRR----KHFTVNRPF
            pse   EEGTEAAAATGAVVRMKRSIVSLTEPIEFHADHPF
            per   EEGTEAAAATGAVVRMKRSIVSLAEPIEFHADHPF
            wil   EQGTEAAAATGFVVRKKRAIVSLTEPVEFFADHPF
            moj   ELGTEAAAATAAVVRMKRSVISIEQPIQFHADHPF
            vir   ELGTEAAAATVFRIMPVLAFRR----KKFLATHPF
            gri   EHGTEAAAATAMVMCFASMPMFQPEPIRFHAEHPF
                  * ********              .   *  . :**

Spn42Db     mel   EVGTEAAAATGEPAKKKGS-KTFGNLKASYI-----
(CG9454)    sim   EVGTEAAAATAAVATFRSM-PAREVPPKVFHANRPF
            sec   EVGTEAAAATAAVATFRSM-PARESPPKVFHANRPF
            yak   EVGTKAAAATVTTPMVFYL-PVILSLQIENS-----
            ere   EVGTEAAAATAVVATFRSM-PPPQGSPKVFHANRPF
            ana   ELGTEAAAATAVVLMVRSL-PAP-EPHQVFIANRPF
            pse   EVGTEAAAATAAVMVMRSLPATPVDRPKAFHANRPF
            per   EVGTEAAAATAAVMVMRSLPATPVDRPKAFHANRPF
            wil   ELGSEAAAATGKFLNMKGYDCDG-------------
            moj   EEGTEAAAATAMLVYFEMMTPTED-EPKIFHADHPF
            vir   EEGTEAAAATAAVMMMRSAPAPESMPEVFHAN-HPF
            gri   EEGTEAAAATALVVKMRSAPLEPKIQIFHAN--HPF
                      **:*****       :

Spn42Dc     mel   EAGSEAAAVSFMKIVPMMLNMNKKLFKADHPF
(CG9455)    sim   EAGSEAAAVSFMKIVPMMLNMNKKLFKADHPF
            sec   EAGSEAAAVSFMKIVPMMLNMNKKLKFADHPF
            yak   EAGSEAAAVSFMKIVPMMLNMNTKHFKADHPF
            ere   EAGSEAAAVSFMKIVPMMLNMNKKLFKANHPF
            ana   EAGSEAAAVSFMKIVPMMLNMNKKQFKADHPF
            pse   EAGSEAAAATFMKIVPMSLNMSKKLFKVDHPF
            per   EAGSEAAAATFMKIVPMSLNMSKKLFKVDHPF
            wil   EAGSEAAAVTFMKIVPMMLNMNKKSFKVDHPF
            moj   EAGCEAAAATYLKIVPMSMIMHQKQFKVDHPF
            vir   EAGSEAAAVTFLKVVPMSLNMHKKTFKVDHPF
            gri   EAGCEAAAVSVLKVVPMSLNMNKETFKVDHPF
                  ***.****.: :*:*** : *  : **.:***

Spn42Dd     mel   EEGAEAAGATSVAVTNRAGFS-----TFLMADHPF
(CG9456)    sim   EEGAEAAGAT-VAVTNRAGFS-----TFLMADHPF
            sec   EEGAEAAGATSVAVTNRAGFS-----TFLMADHPF
            yak   EEGAEAAAATKIVVYPLSAHP---IWMDFNVDHPF
            ere   EEGAEAAGATSVAVTNRAGFS-----MFLAADHPF
            ana   EEGAEAAAATVAMVSPRSGFS-----QSFVADHPF
            pse   EEGAEASAATYGMITNRSSFT-----MVLSFDHPF
            per   EEGVEASAATCTCDLLIRPDYGSMIILTYINVYIF
                  ***.**:.**      :    .  :       : *
```

Spn42De (CG9460)

```
mel   EKGTTASGATFIKVSVESLTIGEEVFEFIADHPF
sim   EKGTTASGATFVKVSVESLLIGEEVFEFIADHPF
sec   EKGTTASGATFVKVSVESLLIGEEVFEFIAYHPF
yak   EKGTSASGATYVNAAVESLLIGEQVFEFTADHPF
ere   EKGTTASGATFVQVAVESLLIGEEVFEFSADHPF
ana   EKGTTASGATFVKAELESLVIGEQTVEFVADHPF
pse   ENGTTASAATAVKFSLESAFMG-EVQQFTADHPF
per   ENGTTASAATAVKFSLESAFMG-EVQQFTADHPF
wil   EKGTTATGASFSKVVLELEVIGEVTYNFTVDHPF
      *:**:*:.*:  :  :*   :*  . :* . ***
```

Spn43Aa (CG12172)

```
mel   EIGCEAAGASYAAGVPMSLPLDPKTFVADHPF
sim   EIGCEAAGASYAAGVPMSLPLDPKTFVADHPF
sec   EIGCEAAGASYAAGVPMSLPLDPKTFVADHPF
yak   EIGCEAAGASYAAGVPMSLPLDPKTFVADHPF
ere   EIGCEAAGASYAAGVPMSLPLDPKTFVADHPF
ana   EIGCEAAGVSYAAGVPMSLPLDPKTFVADHPF
pse   EIGCEAAGVSYAAGVPMSLPLDPKTFVADHPF
per   EIGCEAAGVSYAAGVPMSLPLDPKTFVADHPF
wil   EIGCEAAGISYAAGVPMSLPLDPKTFVADHPF
moj   EIGCEAAGVSYAAGVPMSLPLDPKTFVADHPF
vir   EIGCEAAGISYAAGVPMSLPLDPKTFVADHPF
gri   EIGCEAAGVSYAAGVPMSLPLEPKTFVADHPF
      ******** ************:**********
```

Spn43Ab (1865)

```
mel   EAGVDQPLETGLLKGLFSRSKKFEADHPF
sim   EAGVEQPLETGLLKGLFSRSKKFEADHPF
sec   EAGVEQPLETGFLKGLFSRSKKFEADHPF
yak   EAGLEQPLETGLLKGLFSRSKKFEADHPF
ere   EAGLEQPLETGLLKGLFSRSKKFEADHPF
ana   EAGVEQPLETGVLKGLFSRSKKFEADHPF
pse   EAGVEQPLESGVLKGLFSRSKKFEADHPF
per   EAGVEQPLESGVLKGLFSRSKKFEADHPF
wil   EAGVEPSLEQGVLKGLFSRSKKFEADHPF
moj   EAGVEQPLETGVLKGLFSRSKKFEADHPF
vir   EAGVEQPLESGVLKSFFSRTKKFEADHPF
gri   EAGVEQPLESGLLKGLFSRNKKFEADHPF
      ***::**** *:**.:***.*********
```

Spn43Ac (CG1857)

```
mel   EAGTEASAASYAKFVPLSLPPKPTEFVANRPF
sim   EAGTEASAASYAKFVPLSLPPKPTEFVANHPF
sec   EAGTEATAASYAKFVPLSLPPKPTEFVANRPF
yak   EAGTEASAASYAKFVPLSLPPKPTQFVANRPF
ere   EAGTEASAASYAKFVPLSLPPKPKEFIANRPF
ana   EAGTEAAAASYAKFVPLSLPAKSPEFTADHPF
pse   EAGSEAAAASYAKFVPLSLPVKSREFNADHPF
per   EAGSEAAAASYAKFVPLSLPVKSREFNADHPF
wil   EAGSEASASSYAKFVPLSLPVKSHEFTADHPF
moj   EAGSEAAAATFAKFVPLLLPMKSREFIADHPF
vir   EAGSEAAAASYAKFVPLSLPVKSREFVADHPF
gri   EAGSEAAAASYAKFVPLSLPVQSLEFTADHPF
      ***:**:*:::****** ** :. :* *::**
```

Spn43Ad (CG1859)

```
mel   EDGGNADDSFS--FGDLFRRALPLVINHPF
sim   EEGGNADDSFY--FGDLFRRALPLVINHPF
sec   EEGGNADDSFS--FGDLFRRALPLVINHPF
yak   EEGGNAEDSFS--FGDLFRRALPLVINHPF
ere   EEGGHADDSFS--FGDLFRRALPLVINHPF
ana   EKGGTDGDSFS--FGDLFRRALPLVINHPF
pse   EEGGSAGDSFS--FGDLFRRALPLVINHPF
per   EEGGSAGDSFS--FGDLFRRALPLVINHPF
moj   EAGGEAEQTFVAAFTDLFRSTLSLVINHPF
vir   EAGGEAEQTFVAAFTDLFRSTLSLVINHPF
gri   EQGAEAEQTFFATFTDLFRSTLSVVINHPF
      * *.   ::*   * ****: *.:******
```

Spn47C (CG7722)

```
mel  EFGCEVAPEAEVQPEVLKK-NPDRKFFKADRPF
sim  EFGCEVAPEAEVQPEVLKK-NPDRKFFKADRPF
sec  EFGCEVAPEAEVQPEVLKK-NPDRKFFKADRPF
yak  EFGCEVAPESDVQPEVLKK-NPDRKVFKADRPF
ere  EFGCEVAPESDVQPEVPKK-NPDRKVFKADRPF
ana  EAGCETAPEPDTHIHKTKN-NPDRKIFRADRPF
pse  ESGCETDPDKPARAAAFVQ-NPDRKLFMANRPF
per  ESGCETDPDTPSRAAAFVQ-NPDRKLFMTNRPF
wil  ENGCEVDIENKPGPTVPIVPDAERKIFRANRPF
moj  EAGCTTNIDESTKNRFVKA-NPERKVFVADHPF
vir  EAGCEIDANTPAETSTETS-NPERKVFTADHPF
gri  ESGCGTADHTQGRVAAAKV-NPERKVFKANQPF
     * **    .        :: **.* :::   **
```

Spn53F (CG10956)

```
mel  EATYPREFRVNATKSVMIPMMHEDSKFAFGILGNLKATAVLVPF
sim  EATYPREFRVNAARSVMIPMMHEDRRSP---------------N
sec  EATYPREFRVNAAKSVMIPMMHEDSKFAFGILGNLKATAILVPF
yak  EATYPREFRVSAARKVMIPMMHEDSKFAFGNLATLKASAVLVPF
ere  EATYPRQFRVSAAKEVLIPMMHEDSKFAFGTLGHLKATAVLVPF
ana  GATYDREFRVSSQRTINVPMMHEDSKFAFGDLEKLQATALLLPF
      ***. *** *:***.: : : :****** : .
```

Spn55B (CG10913)

```
mel  EEGTEAAAATGMIMMTRMMTFPLQFQADRPF
sim  EEGTEAAAATGMIMMTRMMTFPLQFQADRPF
sec  EEGTEAAAATGMIMMTRMMTFPLQFQADRPF
yak  EEGTEAAAATGMIMMTRMMTFPLQFQADRPF
ere  EEGTEAAAATGMIMMTRMMTFPLQFQADRPF
ana  EEGTEAAAATGMIMMTRMMTFPIQFQADRPF
pse  EEGTEAAAATGMIMMTRMMTFPLQFQADRPF
per  EEGTEAAAATGMIMMTRMMTFPLQFQADRPF
wil  EEGTEAAAATGMIMMTRMMTFPIQFQADRPF
moj  EEGTEAAAATGMIMMTRMMMLPLQFQADRPF
vir  EEGTEAAAATGIIMMTRMMLMPLQFQADRPF
gri  EEGTEAAAATGMIMMTRMMMLPLQFQADRPF
     ***********:******* :*:********
```

Spn75F (CG32203)

```
mel  ETALTNRTFTLLRQNKKPFVYTTQMMYTEAPMDFFNNDQVRGVMVPF
sim  EKALTNRTFTLLRQNRKPFVYTVEMMYTEAPMEFFNNDQVRGVVVPF
sec  EKALTNRTFTLLRQNRKPFVYTVEMMYTEAPMQFFNNDQVRGVVVPF
yak  EKAMTNRLFSYIRPNRKPFVYKVEMIYTEAPMEFFNEDQVRGVMVPF
ere  EKAMTNRTFRWIRPNRRTFVYRVQMIYIEAPMEFFSGDQCRGVMVPF
     *:* *** *  :* *::.***:*:* * **** **. ** ***:***
```

Spn76A (CG3801)

```
mel  FKDSAFKSKAKIKINNFRVNHGIRFQPILRLEVVDDIDTGKTETFEVNRPF
sim  FKDSAFKSKAKIKINDFRVNHGIRFQPILRLDVVDDIDTGKTEKFEVNRPF
sec  FKDSAFKSKAKIKINDFRVNHGIRFQPILRLDLVDDIDTEKTEKFEVNRPF
yak  FKDSGFSSKPNIKINDFRVNHGVQFEPTQRLNVVEDIDTQNAQTFEVNRAF
     ****.*:**. ***********. *:*  **  * ****   : ***** *
```

Spn77Ba (CG6680)

```
mel  EQGTTAGAVTEAALANKATPPKFLLNRPF
sim  EQGTTAGAVTEAALANKATPPKFLLNRPF
sec  EQGTTAGAVTEAALANKATPPKFLLNRPF
yak  EQGTTAGAVTEAALSNKATPPKFQLNRPF
ere  EQGTTAGAVTEAALANKATPPKFQLNRPF
ana  EQGTTAGAVTAAALINKATPPKFLLNKPF
pse  EQGTTAGAVTAAVLANKATPPKFQLNKPF
per  EQGTTAGAVTAAVLANKATPPKFQLNKPF
wil  EKGTTAAAVTSASLVNKATPPKFVLNKPF
moj  EKGTTAAAVTSAVLSNKSSPPKFYLNRPF
vir  EKGTTAAAVTASVLSNKSTPPKFQLNRPF
gri  EQGTTAAGVTVSSLSNKSTPPKFHLNRPF
     *:****..** : * **::**** **:**
```

Spn77Bb (CG6663)

```
mel  VDEEGLPNAVPQKSSGKN---NIKFHVNRPF
sim  VDEEGLPDAVPQKSSGTN---NIKFHMTRPF
wil  VDEKGTAAAAFSAATLSNKAAPPKFMINKPF
     ***:* . *. . :: .*     ** :.:**
```

Spn77Bc (CG6289)

```
mel   EQFYNAGGSPAGKVEMMVQTGKYAYVNNVKGLQADVLELPF
sim   EQFYDSSGKPAGKVNMMVQTGKFAYIRSIKRLDADFLMLPF
sec   EQFYDSSGKPAGKVDMMVQTGKFAYIRSIRRLDADFLMLPF
yak   EQFYNDDGSPAGKVHMMVQTGKFAYAKNVKDLQADVLELPF
      ****: .*.*****.*******:** ..:: *:**.* ***
```

Spn88F (CG12807)

```
mel   YHTDIVAAAASLKLGPTLRLMRKQLKPR
sim   ----- RTAASLKLGPTLRLMRKQLKPR
sec   YHTDIVAAAASLKLGPTLRLMRKQLKPR
yak   YHTDIVAAAASLKLGPTLRLMRKQLKPR
ere   YHTDIVAAAASLKLGPTLRLMRKQLKPR
ana   YHTDIVGAAASLKLGPTLRLMRKQLKPR
pse   YHTDIVAAAASLKLGPTLRLMRKQLKPR
per   YHTDIVAAAASLKLGPTLRLMRKQLKPR
wil   YHTDIVAAAAALKLGPTLRLMRKQLKPR
moj   YHMDIVAAAASLKLGPTLRLMRKQLKPR
vir   YHMDIVAAAASLKLGPTLRLMRKQLKPR
gri   YHMDIVAAAASLKLGPTLRLMRKQLKPR
      **:***:*********************
```

Spn88Ea (CG18525)

```
mel   EEGSTAAAATVLFTYRSARPVEPAKFECNHPF
sim   EEGSTAAAATVLFTYRSARPVEPAKFECNHPF
sec   EEGSTAAAATVLFTYRSARPVEPAKFECNHPF
yak   EEGSTAAAATVLFTYRSARPVEPAKFECNHPF
ere   EEGSTAAAATVLFTYRSARPVEPAKFECNHPF
ana   EEGSTASAATVLFTYRSARPVEPAKFEANHPF
pse   EEGSTAAAATVLVSYRSARPIEPTKFECNHPF
per   EEGSTAAAATVLVSYRSARPIEPTKFECNHPF
wil   EEGSTAAAATVLVSFRSARPIEPTKFECKHPF
moj   EEGSTAAAATVLVSFRSARPVEPTKFECNHPF
vir   EEGSIAAAATVLVSFRSARPIEPTKFECNHPF
gri   EEGSTAAAATVLVSFRSARPAEPTKFECNHPF
      **** *:*****.::***** **:***.:***
```

Spn88Eb (CG6687)

```
mel   EVGSTAAAATILLVSRSSRQPDPTKFNCNHPF
sim   EVGSTAAAATILFVSRSARQPDPTKFNCNHPF
sec   EVGSTAAAATILFVSRSARQPDPTKFNCNHPF
yak   EVGSTAAAATILFASRSARQPDPSKFNCNHPF
ere   EVGSTAAAATILFVSRSARQPDPTKFNCNHPF
      ************:.***:*****:********
```

Spn100A (CG1342)

```
mel   EGGSSANSLSAATMQARTPSV---ESTVLPVPEPEPELP-GVERFEVNRPF
sim   EGGSSANSLSAATMQARTPSV---ESTVLPVPEPEPELP-GVERFEVNRPF
sec   EGGSSANSLSAATMQARTPSV---ESTVLPVPEPEPELP-GVERFEVNRPF
yak   EGGSSANSLSAATMQARTPSV---ESTVLPVPEPEPELP-GVERFEVNRPF
ere   EGGSSANSLSAATMQARTPSV---ESTVLPVPEPEPELP-GVERFEVNRPF
ana   EGGSSTNALSAANMQARTPSV---ESTVLPVPEPEPE-P-GVERFEVNRPF
pse   EGGSSANALSAAT-QARSPQAAEDSASVLPVPEPEPE-P-GVERFEVNRPF
per   EGGSSANALSAAT-QARSPQAAEDSASVLPVPEPEPE-P-GVERFEVNRPF
wil   EGGSSANALSAANMQARSPLA---SATELPVPEPEPE-P-GVERFEVNRPF
moj   EDGSSTNSLSAGNIQARSPIAAE-AAAVLPVPEPEPE-P-GVERFDVNRPF
vir   EGGSSANSLSAATMQGRSPIAAE-AAATLPVPEPEPE-P-GVELFDVNRPF
gri   EGGSSANSLSAATTQARSPLFIL----QEPEPEPEPE-PEGVERFEVNRPF
      *.***:*:***.. *.*:*          * ****** * *** *:*****
```

Additional Figure 1: Multiple alignment of Serpin RCL sequences.

Alignment annotation: * Identical residues, : closely similar residues, . broadly similar residues. Inhibitory serpins, indentified in red, like Spn27A (CG11331), are

characterised by a consensus sequence of small side-chain residues: E[EKR]G[TSG][ET][AGS][YAGS][AGS][VAGS][TS]. These residues form a flexible hinge region, 17-8 residues N-terminal to the putative protease cleavage site residues, P1/P1´, marked in green. Both inhibitory serpins and non-inhibitory serpin folds have conserved PF residues at the C-terminal “shutter region” of the RCL. Note that the *Spn42Da* orthologues contain a mixture of several splice variants and the putative P1/P1´sites are unmarked. *Spn100A* represents a novel protein fold of unknown function, with two stretches of homology to the serpin-fold, one of which contains a putative RCL with partial conservation of the inhibitory serpin flexible hinge-region residues.
