# Supplementary material for: Identification and analysis of serpin-family genes by homology and synteny across the 12 sequenced Drosophilid genomes
Source: BMC Genomics. 2009 Oct 22;10:489. doi: 10.1186/1471-2164-10-489 (PMC2770083; doi:10.1186/1471-2164-10-489)
Supplement: Additional file 3 — Multiple sequence alignment of Drosophilid Spn43Ac orthologues. Alignment of the Spn43Ac orthologues indicate that a long N-terminal extension is conserved across the Drosophilid species, although sequence conservation is poor, and the length varies between 69 and 145 amino acids. [file 1471-2164-10-489-S3.PDF]

```
                     10        20        30        40        50        60
                      |         |         |         |         |         |
dmel         maskvsilllltvhllaaqtfaqeliawqr------------------------------
dsim         maskvsilllltvhllatqtfaqeltawqr------------------------------
dsec         maskvsifllltvhllaaqtfaqeltawqr------------------------------
dere         maskttllllltisllaahsfaqefsawqr------------------------------
dyak         maskatilllltty--ghllaaqeyhgwsr------------------------------
dana         maskcmillallvpllnaqtfnqpgypgfg------------------------------
dpse         maskciilll-dgplp-----rgcsiplpgd-------------irlaspstara-----
dper         maskciillllmgpyl-----vaaqsyyrgt-------------yasrlrqrpel-----
dwil         mtckysilllvasvsagrqnrrpqqqpvapv-------------arqdlvpraaa-----
dmoj         mpnkysllllgs-clslclfltpatgngqri----------idygnivfsrrs-------
dvir         mllllssylilslslclci--gpavsssifagv--------iinsrlpfsiqsrsyvgp-
dgri         msckysilllfslclflflfsgsvastgiyarvnnvvytnnawlsnqpidirsrsgarav
Consensus    MasK siLLLl   l
Prim.cons.   MASK3SILLLLTV2LLAAQTFAQELSAWQRA2VNNVVYTNNI33SR2P2SIRSR22222V

                     70        80        90       100       110       120
                      |         |         |         |         |         |
dmel         --qqqqqqqqqlqlqqqlllqqqqhqrnprpelglrs-----------------------
dsim         --qqqlqlqqqqrqqqq-----qqqqlnprpelglrsrm---------------------
dsec         --qqqlqlqqqqrqqqq-----qq--lnprpelglrs-----------------------
dere         --hqqplqqqqqqlpr-----------nprpelglrs-----------------------
dyak         --ppqqqqpr-----------------ssrpelglra-----------------------
dana         --lgarfgggq-----------------vvpqqdlqa-----------------------
dpse         --qgqvlpqpqgppdnq-----rpleevqppgnsqerq--------------------pq
dper         --rgkscrrprarqttn-----ghwrrssrretarrds--------------------pk
dwil         --aapsvpaaaakgandlqrpiggwdqqwnnnnvqtnq--------------------qy
dmoj         --sfdnqiqsrsrvaapaqnristvsaaasppniqqqq--------ypaqpla----pve
dvir         -pdlhdlqrpqgevmplaqetwsdqriaaashtgqqtwnvqrnvaevptaqqqqqhqpip
dgri         kpteqdqqnpqqdpqltaqqswnsqrsaqrpetsagtvnl------vpktqlqq---pns
Consensus        q     q                  rpe
Prim.cons.   KPQQQ4QQQ2QQRQQQQAQ552QQQR2NPRPELGLRSQN2QRNVAEVP33QLQQQHQPP6

                    130       140       150       160       170       180
                      |         |         |         |         |         |
dmel         lpgnpwt-qnnqeaisdvvavdltkrepvtpppnrPP--PVFSYMDRFSSELFKEIIKSQ
dsim         ypenrwnnqnnqeaisdvvavdltksepvtppptrPP--PVFSYMDRFSSELFKEIIKSQ
dsec         lpgnrwi-qnnqeaisdvvavdlskrepvtppptrPP--PVFSYMDRFSSELFKEIIKSQ
dere         ----qgtnqytkeptsdvvmvdltkhepanppptrPP--PVFSYMDRFSGQLFDQIIKSQ
dyak         -qntnprtqdnqhpisdvvavdlskpeptnapptrPP--PAYSYMDRFSAELFKQIMKSQ
dana         -------qedirvpsaprqrnggqsrnpaptaptrAPVIPKTSFMDRFSSKLYAKIAPAQ
dpse         npnsqippqrqpanvlgane--vplptvsnsxptrPP--AKQSFKDRFSSKLFQPIASRS
dper         iptaryrprdsrpmcwvpmr--crcpr-sptvptrPP--AKQSFKDRFSSKLFQPIASRS
dwil         gptandsprvqgvdesgsgs--glsqppvstsptrPP--AQKNYMEQFSSKLFQQIATRA
dmoj         apdasrtpsvtvlgidgsadqsaagqsrgpsgpirPP--AQFNYRERFSSTLFQPISRNN
dvir         asasasapaaspevivngfnq-aagrpassagptrPP--AQYNYRERFSSVLFQPISRSN
dgri         apalatapavtv-lgvdrpnq-asvqpsssssptrPP--AHYNYRERFSSKLFQPIARNN
Consensus     p     p                   p    PTRPP  a  s%m#RFSS LFq I
Prim.cons.   AP42RWTPQ3NQE2ISDVVAVDL2KREP24PPPTRPPVI2VFSYMDRFSSKLFQPIAKSQ
```

```
                    190       200       210       220       230       240
                      |         |         |         |         |         |
dmel         SQQNVVFSPFSVHALLALIYGASDGKTFRELQKAGEFSKNAMAVAQDFESVIKYKKHLEG
dsim         SQQNVVFSPFSVHALMALVYGASDGKTYRELKKAGEFSKNAMAVAQDFESVIKYKMHLEG
dsec         SQQNVVFSPFSVHALLALVYGASDGKTYRELKKAGEFSKNAMAVAQDFESVIKYKMHLEG
dere         GQQNVVLSPFSVHALLALIYGASDGKTFRELQKAGEFSKNAMAVAQDFESVIKYRMHLEG
dyak         SQQNVVFSPFSVQALLALIYGASSGKTFRELQKAGEFSKNAMAVAHDFENVIKYKMHLEG
dana         AGSNFVYSPVSVHSILALIYGTSFGKTRRELKSAGEFVDDQIDVAMIFEKLIKFRSDLGN
dpse         QRQNLVFSPVSVHALLGMIYGASEGRTAQELQQAGEFGPDPQAVGQDFRQLIKQRRQLQS
dper         QRQNLVFSPVSVHALLGMIYGASEGRTAQELQQAGEFGPDPQAVGQDFRQLIKQRRQLQS
dwil         QQKNIVYSPAMVHSQLAMLYIVSHGQTFEELQQAGIFSVDTTKVSQDFLSLLSLQ-QLQN
dmoj         ANRNVVYSPSSVHAMLAMLYGVSAGETATELRSAGQFDQNQLTTAMDFQRVRKLERELQN
dvir         GQQNVVYSPATMHAMLGLLYGVSSNETAAELQRVGQFGNKQLDVAIEFEQVRRTESQLPN
dgri         ARKNVVFSPASMHSMLGLLYSVSSGQTSDELQRAGNFDIGKIEVAMDFKNVDQQHKKLVN
Consensus     qqNvV%SP SVHa$La$ YGaS G T  ELq AG#F  #  aVaqDFe vik    L
Prim.cons.   SQQNVVFSPFSVHALLALIYGASDGKT2RELQKAGEFSKNAMAVAQDFESVIKY2MHLE2

                    250       260       270       280       290       300
                      |         |         |         |         |         |
dmel         ADLTLATKVYYNRELGGVNHSYDEYAKFYFSAGTEAVDMQNAKDTAAKINAWVMDTTRNK
dsim         ADLTLATKXYYNRELGGVXPSYDEYAKFYFSAGTEAVDMQNGKDTAARINAWVMDQTRNK
dsec         ADLTLATKVYYNRELGGVNPSYDEYAKFYFSAGTEAVDMQNGKDTAARINAWVMDQTRNK
dere         ADLTLATKVYYNQELGGVNPSYDAYAKFYFSAGTEAVDMQNGKDTSARINAWVMDQTRSK
dyak         TDLTLATKVYYNQELGGVNHSYDAYAKFYFSSDTEAVDMQNGKDTAARINAWVMDKTRSK
dana         VELKMATKLYHNQLKGGAYPGFPEFSQFYFNTADEAVDMTRAKDTSEKINFWVSDSTDGK
dpse         AELTMASRMFYNKNMGGINHDYPEYAEYYYSSGIEPVDMGRSRETAGWINAWVSDKTRNK
dper         AELTMASRMFYNKNMGGINHDYPEYAEYYYSSGIEPVDMGRSRETAGWINAWVSDKTRNK
dwil         AEIIVASKVLYNPALGQPNERFPKYALTYFNTEIETFNPQDPRNTANAINGWVRDRTKNT
dmoj         AQLIVANKLFYNHELAMVNPDYAHYAHLYFNSEIEGVNMKRSANTASRINAWAADATRNI
dvir         AQLIVANKLYYNREIDELNPRYLAFASQYYGSETEAVNMRKSRDTAAKINAWASDATRGI
dgri         TRLIVANKLYYNRELSAPNDRYEAFALEYYNSEIEAVDMKKPRNTAAEINQWVSRATNKI
Consensus    a#Lt A K %YN #$gg N  Y  %A  Y%ss  EaV#M    #TAa INaWV D Trnk
Prim.cons.   ADLTLATKVYYNRELGGVNPSYDEYAKFYFSSGTEAVDMQN2KDTAARINAWV2D3TRNK

                    310       320       330       340       350       360
                      |         |         |         |         |         |
dmel         IRDLVTPTDVDPQTQALLVNAVYFQGRWEHEFATMDTSPYDFQHTNGRISKVAMMFNDDV
dsim         IRELVTPADVDPQTQALFVNAVYFKGRWEHEFATMDTSPSDFQHSNGRISKVAMMYNDDV
dsec         IRELVTPADVDPQTQALLVNAVYFKGRWEHEFATMDTSPSDFQHSNGRISKVAMMYNDDV
dere         IRELVTSGDIDPQTQALLVNAVYFKSRWEHEFAIMDTAPSDFHHSNGKTSQVAMMYNDDV
dyak         IRELVTPGDMDPQTQALLVNAVYFQGRWEHEFAIMDTSPYDFHHSNGRTSKVAMMYNDDV
dana         IRNLAAPSDITEQTEALLVNAIYFKGRWENEFATMDTQPSNFKHSDGRISSVAMMYNDDV
dpse         IRELVTPSDIDGQTEAMLVNAIYFKARWATEFSATDTISGKFRRGSGAPSNVAMMFNDDV
dper         IRELVTPSDIDGQTEAMLVNAIYFKARWATEFSATDTISGKFRRGSGAPSNVAMMFNDDV
dwil         IKQLITQSEIDDQTQAILLNAIYFKARWANEFSTRDTMPAKFRMGNGAAINVAMMYNDDV
dmoj         IRDLVSPNDIDDETQALLVNAIYFKARWANEFSAMDTTPDKFRVNSNKAVTVAMMYNDDV
dvir         IRDLVQPSDIDEQTQALLVTAIYFKARWANEFSEMDTTAEKFRMGNNAAITVPMMYNDDL
dgri         IRELVSPSDIDEQTEAMMVDAIYFKARWANEFSAMDTTPAKFRLNGVTPITVPMMYNDDI
Consensus    IR#LVtPsD!D QT#A$LVNA!YFKaRWa EFs mDT p kFr  ng  s VAMM%NDDV
Prim.cons.   IRELVTPSDIDPQTQALLVNAIYFKARW22EF2TMDTSPSKFRHSNGRISKVAMMYNDDV

                    370       380       390       400       410       420
                      |         |         |         |         |         |
dmel         YGLAELPELGATALELAYKDSATSMLILLPNETTGLGKMLQQLSRPEFDLNRVAHRLRRQ
dsim         YGLAELPELGATALELAYKDSATSMLILLPNQTTGLAKMLQQLSRPEFDLNYVAHRLRRQ
dsec         YGLAELPELGATALELAYKDSATSMLILLPNQTTGLAKMLQQLSRPEFDLNYVAHRLRRQ
dere         YGLAELPELGATVLELAYKDSAASMLILLPNQTNGLAKMMQQLSRPEFDLNRVAHSLRRQ
dyak         YGLAELPELGATALELAYKDSATSMLILLPNQTNGLAKMMQQLSRPEFDLNRVAHRLRRQ
dana         YSLADIPELGASALGLNYRDSNISMLILLPKQVNGLRALEAQLADPQFDLNRIAARLQRQ
dpse         FGYADLPDLGATALEMPYADSEVSMLILLPYQVDGLAQLEQQLARPQNDLNRIAARLRQE
dper         FGYADLPDLGATALEMPYADSEVSMLILLPYQVDGLAQLEQQLARPQNDLNRIAARLRQE
dwil         FDYAELPDLHATALEMPYAGTQISMLIILPNQVNGLTQLERQLARPEYDLNAIAARLRRE
dmoj         YAYAELPDLDAVALELPYAGTEVSMLFVLPNQVDGLPQLERQLE--RTDLNQIAARLRRE
dvir         FDIAELPELDATALELPYAGTPISMLIILPNQVNGLAQLERQLE--RHDLNQIAARLHRD
dgri         FAYAQLPELDATALELPYVDNDASMLLILPNQPNGLAQLERNLASTNHDLNAIAARLRRE
Consensus    %g A#LP#LgATALE$pY ds  SMLIlLPnQvnGLaq$eqQL rp# DLN !AaRLRR#
Prim.cons.   YGLAELPELGATALELPY2DSATSMLILLPNQVNGLAQLEQQL2RPEFDLNRIAARLRRQ
```

```
                    430       440       450       460       470       480
                      |         |         |         |         |         |
dmel         SVAVRLPKFQFEFEQDMTEPLKNLGVHQMFTPNSQVTKLMDQPVRVSKILQKAYINVGEAGTEASAAS
dsim         PVAVRLPKFQFEFEQDMTQPLKDLGVHQMFTPNSQVTKLLDHPVRVSKILQKAYINVGEAGTEASAAS
dsec         PVAVRLPKFQFEFEQDMTQPLKDLGVHQMFTPNSQVTKLLDHPVRVSKILQKAYINVGEAGTEATAAS
dere         SVAVHLPKFQFEFEQDMTQPLKDLGVQQMFTPNSQVTKLLDQPVRVSKILQKAYINVGEAGTEASAAS
dyak         SVAVRLPKFQFEFEQDMTQPLKDLGIHQMFTPNSQVNKLLDHPVRVSKILQKAYINVGEAGTEASAAS
dana         NVLVRLPKFRIEFDQDMTQPLKQMDVQEMFGPKSQIKTMLNDRVRVEKILQKAFIDVNEAGTEAAAAS
dpse         TVTIRIPKFRIQFEQDMTEPLQQLGVREMFTRSSQVTKMLDRPVRVSKILQKAFLNVNEAGSEAAAAS
dper         TVTIRIPKFRIQFEQDMTEPLQQLGVREMFTRSSQVTKMLDRPVRVSKILQKAFLNVNEAGSEAAAAS
dwil         TVTVRIPRFRIEFEQDMTEPLQRLGVKEMFTSRSQVDAMLDKPAMVSKIQQKAFIDVNEAGSEASASS
dmoj         MVAVRLPKFRIEFEQDMTLPLQELGVRRMFTANSQVNTILMRPVKVSKILQKAFIDVNEAGSEAAAAT
dvir         MVTVRLPKFRIEFELDMTGPLQQLGVRRMFTPKSQVDAMLLQPVRVSKILQKAFIDVNEAGSEAAAAS
dgri         MVTVRVPKFRIEFEQDMSGPLQELGVRRMFTKQSEVDAMLMQPVRVSKVLQKAFIDVNEAGSEAAAAS
Consensus     V VRlPKFriEFEQDMT PLq#LGV  MFTp SQV k$Ld PVRVSKILQKA%I#VnEAGsEAaAAS
Prim.cons.   3VAVRLPKFRIEFEQDMTQPL22LGVRQMFTPNSQVTKMLDQPVRVSKILQKAFINVNEAG2EAAAAS

                    490       500       510       520       530       540       550
                      |         |         |         |         |         |         |
dmel         YAKFVPLSLPPKPTEFVANRPFVFAVRTPASVLFIGHVEYPTPMSV-----------------
dsim         YAKFVPLSLPPKPTEFVANHPFVFAVRTPTSVLFIGHVEYPTPVSV-----------------
dsec         YAKFVPLSLPPKPTEFVANRPFVFAVRTPTSVLFIGHVEYPTPVSV-----------------
dere         YAKFVPLSLPPKPKEFIANRPFVFAIRTPTSVLFIGHVEYPTPMSVKN-KPNFDFYRN-----
dyak         YAKFVPLSLPPKPTQFVANRPFVFAIRTPTAVLFIGHVENPTPMSVKNPEPYSDIYRN-----
dana         YAKFVPLSLPAKSPEFTADHPFVFAIRTPTSVLFIGHVEHPTPLTASPEGAGRGRQRSFSSRQ
dpse         YAKFVPLSLPVKSREFNADHPFVFALRTPDSVLFIGHVLQPTAMA------------------
dper         YAKFVPLSLPVKSREFNADHPFVFALRTPDSVLFIGHVLQPTAMA------------------
dwil         YAKFVPLSLPVKSHEFTADHPFIFAIRSPNAVLFIGHVVQPTQVA------------------
dmoj         FAKFVPLLLPMKSREFIADHPFLFAIRTPESVLFIGHVVQPPQINARQ---------------
dvir         YAKFVPLSLPVKSREFVADHPFVFAIRTPDSVLFIGHVVQPPQVSGRQ---------------
dgri         YAKFVPLSLPVQSLEFTADHPFLFAIRTPETVLFIGHVVQPLQATRSQ---------------
Consensus    YAKFVPLSLP Ks EF A#hPF!FA!RTP sVLFIGHV qPt
Prim.cons.   YAKFVPLSLP2KS2EFVADHPFVFAIRTPTSVLFIGHVEQPTPMSV3Q23P33D3YRNFSSRQ
```

Alignment annotation:

Residues conserved for 80 % or more (upper-case letters) : 195 is 35.39 %

Residues conserved for 50 % and less than 80 % (lower-case letters) : 87 is 15.79 %

Residues conserved less than 50 % (white space) : 233 is 42.29 %

IV conserved positions (!) : 5 is 0.91 %

LM conserved positions ($) : 7 is 1.27 %

FY conserved positions (%) : 8 is 1.45 %

NDQEBZ conserved positions (#): 16 is 2.90 %

Residues of the N-terminal extension are printed in lower case, with serpin core residues in UPPER CASE. The putative scissile bond marked in bold (**rP**). The consensus flexible hinge region residues of inhibitory serpins and the PF residues at the C-terminal RCL "shutter region" are marked in RED. The putative protease cleavage site in the RCL, P1/P1´, is marked in green.
