# Supplementary material for: Identification and analysis of serpin-family genes by homology and synteny across the 12 sequenced Drosophilid genomes
Source: BMC Genomics. 2009 Oct 22;10:489. doi: 10.1186/1471-2164-10-489 (PMC2770083; doi:10.1186/1471-2164-10-489)
Supplement: Additional file 4 — Multiple sequence alignment of Drosophilid Spn100A orthologues. The alignment of Spn100A orthologues indicates that this gene is a serpin-related fold showing two blocks of serpin homology separated by an insertion of 294 amino-acids. [file 1471-2164-10-489-S4.PDF]

```
dmel            mkavlcaifvtllaaigqglptq-----------ledenhgsfagqvsqlialqllkfn
dsim            mkavlcaifvtlltavgqglptq-----------ledenqgsfagqvsqlialqllkfn
dsec            mkavlcaifvtllaavgqglptq-----------ledenqgsfagqvsqlialqllkfn
dyak            mkavlcaifvtllaafghglptq-----------lehenqgsfagqvsqlialqllkfn
dere            mkavlcaicvtllaafghglpaq-----------ladenqgsfagqvsqlialqllkfn
dana            mkavlcavfigflallvqglptq-----------gge----sfagrtsqlialqllkfn
dpse            mkavlcvlpllaalvcslptaek-----------essskgasfasatsqliamqllkfn
dper            mkavlcvlpllaalvcslptaek-----------egsskgasfasatsqliamqllkfn
dwil            mkvvvclfplllllsswvsslpta----------dkealpntftsdasqllaiqllkhn
dmoj            mkavlhllsllagasivyslpslgarenalp---------ssfaseasqliatqllkhn
dvir            mkaalcllpllacanivcslppptkedalp---------smfaseasqliatqllkhn
dgri            mkaalglipllgcisivcslpnpqtraeagpgageddkvpatfaseasqliamqllkhn
                **..     :          .                     *:. .***:* ****.*

dmel            kdidanqvhSPLGVASILAMLAEASEGDTYSEFEQVFGYPKDRTKLRDAYKRILgsyqnr
dsim            kdidanqvhSPLGVASILAVLAEASEGDTYSEFEQVFGYPKDRTKLRDAYKRILgsyqnr
dsec            kdidanqvhSPLGVASILAVLAEASEGDTYSEFEQVFGYPKDRTKLRDAYKRILgsyqnr
dyak            kdidanqvhSPLGVASILAVLAEASEGDTYSEFQQVFGYPKDRTKLRDAYKRILgsyqnr
dere            kdidanqvhSPLGVASILAVLAEASEGDTYSEFEQVFGYPKDRTKLRDAYKRILgsyqnr
dana            kdidsnqvhSPLGVASILAVLAEASEGDTFQEFQQVFGYPKDRTQLRDIYQRILgsyqnr
dpse            kdidanqvhSPLGVTSILGVLAEASEGETYAEFTKVFGYPKDRSQLRDVYRRVLgryqnr
dper            kdidanqvhSPLGVTSILGVLAEASEGETYAEFTKVFGYPKDRSQLRDVYRRVLgryqnr
dwil            kdidanqvhSPLGVATILAVLAEASEGATYDEFNTVFGFPKDRSALRESYKRILgnyrnr
dmoj            kdidanqvySPLGVATILATLAEGADGETYAEFGQVFGFPKERQQLRAAFEQALavfqkr
dvir            kdidanqvhSPLGVASILAVLAEAAEGETYAEFGQVFGFPKERQDLRAAFQRILgsyqnr
dgri            kdidsnqvhSPLGVATILAVLAEAAQGETYEEFSQVFGFPKERGDLRAAFQRILanyqnr
                ****:***:*****::**. ***.::* *: **  ***:**:*  **  :.: *. :::*

dmel            daavalpsfqtwlyiyrnnsareefkdllekhyyvdvkdisrqeydwnepntslqlseek
dsim            daavalpsfqtwlyiyrnnsareefknllekhyyvdvkdisrqeydwnepntslqlseek
dsec            daavalpsfqtwlyiyrnnsareefknllekhyyvdvkdisrqeydwnepntslqlseek
dyak            daavalpsfqtwlyiyrnnsareefkdllekhyyvdvkdisrqeydwnepntslqlseek
dere            daavalpsfqtwlyiyrnnsareefkdllekhyyvdvkdisrqeydwnepntslqlseek
dana            daavalpsfqtwlyiyrnnsareefkdvlqknyfvevkdinrqeydwsepntslqleeva
dpse            daavalpsfqtwfyiyrnhsareefkellqqhyfvevkdinrkdydwnepntslqlqpeg
dper            dsavalpsfqtcfyiyrnhsareefkelrqqhyfvevkdinrkdydwnepntslqlqpeg
dwil            daavalpsfqtwlyiyrnnsaredykqlleqnyyvevkdinrqdydwsepntslqleeag
dmoj            dtvdalpsfqtwfyvyrnnsvredykqllqrhyyaevkdinreesdwqep-ssltlegsg
dvir            daavalpsfqtwfyvyrnnsvredykqllqrhyyvevkdinrqeydwsepntslqlegsg
dgri            dsavtlpsfqtwfyvyrnnsvfedykqllqsyyyvdvkdisrqdydwsepntllqldan-
                *:. :****** :*:***:*. *::*:: :  *:.:****.*:: **.** : * *.

dmel            dntdstegste------qsnskdvigfetlkriklddde---dipaipsdgygqeiidkea
dsim            dntdstegste------qsnskdvigfetlkriklddde---disaipadgygqeiidkea
dsec            dnadstegste------qsnskdvigfetlkriklddde---dipaipadgygqeiidkea
dyak            dnadstegste------qsnskdvigfetlkriklddde---dipaipadgygqeiidkea
dere            dntdategste------qsnskdvigfetlkriklddedipdipaipadgygqeiidkea
dana            d-----gsnte------psnskdvigfetlkrikldeg---ddvpaipdsygqeiidkea
dpse            sgsdaseageeslssteqsnskdvigfetlkrinlddd----llsvpadtygeevldkea
dper            sgsdapeaaaeaa-----------------------------------------------
dwil            advdirtepsn---------skdvigfetlkrinvddd----vplvttdtygveivnkea
dmoj            tdgdstepsns----------kdvigfetlkrinvddd---apigisedsygeevvnkea
dvir            advdstepsns----------kdvigfetlkrinlddd---aplvps-dtygeevlnkea
dgri            -----nkssng----------kdvigfetlkriklddd---daplatsdnygeevldkea


dmel            skfdrdvddkqyvekpvaqaeaellqkeqkqqatteseles--------qpeetttlsve
dsim            skfdrdvddk--------------------------------------------------
dsec            skfdrdvddkqyvekpvaqaeaellqkeqkqqatteseles--------qpeetttlave
dyak            skfdrdvddkqyvekpvaqaeaellqkeqkqqattvseles--------qpeetttlave
dere            skfdrdvddkqyvekpvaqaeaellqqeqkqqattelelelesles------qpeetttlave
dana            skfdrevddkqyvekpvaqaeadqlqkeqaaatteselesqsep-----eattaaaivae
dpse            skldrdvddkqyvekpvaqaeaeqlqreqaaatttteaeaeattpeaeaa----------
dper            ---aeaaaeavekqekqaia--------------------------aeaaaeaaaeavek
dwil            skfdrevddkqyvekpvalaeavqmlkeqpfttesaeaaat--------tdaaadmekle
dmoj            skfdrevddkqyvekpvalaeaeqlqs-----ereqltteqtat---dadsssssthapv
dvir            skfdrevddkqyvekpvalaeaaeqlqrdqerereqlttelsat---eaasss--tdapv
dgri            skfdrdvddkqyvekpvalaeaqlatea---------------------------avagt


dmel            kqekpdmaaeenpaekqqnkrsdqe----esqiknleenetvqeeeklakimaap-----
dsim            qyekpdmaaeenpaekqqnkrsdqe----esqiknleenetvqeeeklakimaap-----
dsec            kqekpdmaaeenpaekqqnkrsdqe----esqiknleenetvqeeeklakimaap-----
dyak            kqekpdvaaeenpaekqqnkrsdqe----esqiknleenetvqeeeklakimaat-----
dere            kqekpdmaaeenpaekqqnkrsdqe----esqiknleenetvqeeeklakimaas-----
dana            kqekpdmtveenpvekqqnkrsdqengpeesqiknleenetvqeeeklaklvpvdn---a
dpse                  aaagenpvekqqnkrsdneen--qsqnlnleenetvqeeeklrkvvgse-----
dper            qekqaiaaagenpvekqqnkrsdneen--qsqnlnleenetvqeeeklrkvvgse-----
dwil            kpaepamageenladkqqnkreenlqan-------veenetvqedeklnkqpman-----
dmoj            eklekpaeialtvkenvaekqqnkrsdanaeqnpnieenetvqedeklrkllpsdqgnaa
dvir            eklekpaeialtveenvaakqqnkrs--daeqnpndeenetvqedeklrklln-epsngg
dgri            ekpvipddtlivtsgdsdvdirlptkrsgseqqpnveenetvqedekihkqls-----ta
                :              .   .                ********:**: *

dmel            -altagepekvrlplqklenavktaakdg--adeimlaleshlpsvsrvngarslfqqdd
dsim            -altagepekvrlplqklenavktaakdg--adeimlaleshlpsvsrvngarslfqqdd
dsec            -altagepekvrlplqklenavktaakdg--adeimlaldshlpsvsrvngarslfqqdd
```

```
dyak            -altagepekvrlplqklenavksaakdg--adeimlaleshlpsvsrvngarslfqqdd
dere            -altagepekvrlplqklenavktaakdg--adeimialeshlpsvsrvngarslfqrdd
dana            aavtagepekvrlpleklesavkavtkdg--adeimialeshlasvsraygarslfrqdd
dpse            -avtagepekvrlplqklesavntaaskeggadeimlaleshlgavsrvygarslfrqdd
dper            -avtagepekvrlplqklesavntaaskeggadeimlaleshlgavsrvygarslfrqdd
dwil            eqvtagepekvrlplqklenavktavkds--adeimlaleshlsavsrvygarslfrrdd
dmoj            nqvtasepekvrlplqklenavksmvkeg--adeimialeshlssvarvyggrslfrkdd
dvir            nqvtagepekvrlplqklenavksmvkeg--adeimialeshlstvaraysarslfrqdd
dgri            dqvtdgepekvrlplqkletavksmikeg--adeimialeshlsnvaraygarslfrqdd
                  :* .*********:***.**::  ..   *****:**:***  *:*. ..****::**

dmel            itsalsansitgrsagSKSKMLLFNGLYYRGSWANPFYQLRDGSDEFFFMTNEDAVKAPM
dsim            itsalsansitgrsagSKSKMLLFNGLYYRGSWANPFYQLRDGSDEFFFMTNEDAMKAPM
dsec            ITSALSANSITGRSARSKSKMLLFNGLYYRGSWANSFYQLRDGSDEFFFMTNEDAMKAPM
dyak            itsalsansitgrsagSKSKMLLFNGLYYRGSWANPFYQLRDGSDEFFFMTNEDAMKAPM
dere            itsalsansitgrsagSKSKMLLFNGLYYRGSWANPFYQLRDGSDEFFFMTNEDAMKAPM
dana            itsalsansitgrsagSKSKMLLFNGLYYRGSWANPFYQLRDGSDEFFFMTNEDAMKAPM
dpse            itsalsansitgrsagAKSKMLLFNGLYYRGSWATPFYQLRDGSDEFFFMTNEDAMKAPM
dper            itsalsansitgrsagAKSKMLLFNGLYYRGSWATPFYQLRDGSDEFFFMTNEDAMKAPM
dwil            iasalsansitgrsagSKSKMLLFNGLYYRGSWAQPFYQLRDGSDEFFFMTNEDAMQAPM
dmoj            iacalsanaitgrandAKTKMLLFNGLYFHGSWRQPFYQLRDGSDEFFFMTNEDAMKTPM
dvir            iasalsansitgrelgSKSKMLLFNGLYYRGSWAQPFYQLRDGSDEFFFMTNEDAVKTPM
dgri            iasalsansitgravdSKSKMLLFNGLYYRGSWAQPFHQLRDGSDEFFFMTSEDAMKTPM
                *:.*****:****   :*:*********::***  .*:*************.***:::**

dmel            MHARGKFQVADLPQVKARVLSLPYETSRYALCIVLPDETEGLSDVISQLQTSDFLLAKKQ
dsim            MHARGKFQVADLPQVKARVLSLPYETSRYALCIVLPDETEGLSDVISQLQTSDFLLAKKQ
dsec            MHARGKFQVADLPQVKARVLSLPYETSRYALCIVLPDETDGLSDVISQLQTSDFLLAKKQ
dyak            MHARGKFQVADLPQVKARVLSLPYETSRYALCIVLPDETEGLSDVISQLQTSDFLLAKKQ
dere            MHARGKFQVADLSQVKARVLSLPYETSRYALCIVLPDETEGLSDVISKLQTSDFLLAKKQ
dana            MHARGKFQLAELPKLKAKVLSLPYETSRYSLCIVLPDETEGLSDVIDQLETSDFQQAHKS
dpse            MHARGQFLVADLPQLKARVLSLPYETSRYALCILLPDETEGLHDVIAQMQPSDFQLGREK
dper            MHTRGQFLVADLPQLKARVLSLPYETSRYALCILLPDETEGLHDVIAQMQPSDFQLGREK
dwil            MHTKGKFYVADLPQLKARVLSLPYENEKYGLSIVLPNETEGLSEVIAQMQPSDFKYARDH
dmoj            MHARGKFNVSDLPHLKARLLSLPYENDQYSLCIVLPNEPEGLSEVIAQLEPSDYKYAREH
dvir            MHARGKYNVTDLPHLKARLLSVPYENAQYSLCIVLPNEPEGLSEVIAQLQPSDYKYAREH
dgri            MHAQGKFNVAELPHLKARLLALPYENAQYSLCIVLPNEAEGLSEVIAQLQPSDYRFANEH
                **::*:: :::*.::**::*::***. :*.*.*:**:*.:** :** :::.**:  ...

dmel            FQMKELHISMPKFQVEETSRSEAMLKQMGLKKVFSRTEAQLSLLSEDPDVHVDEIVQFVN
dsim            FQMKELHISLPKFQVEETSRSEAMLKQMGLKKIFSRTEAQLGLLSEDPDVHVDEIVQFVN
dsec            FQMKELHISLPKFQVEETSRSEAMLKQMGLKKIFSRTEAQLGLLSEDPDVHVDEIVQFVN
dyak            FQMKELHVSLPKFQVEETSRSEAMLKQMGLKKVFSRTEAQLGLLSEDPDVHVDEIVQFVN
dere            FQMKELHVSLPKFQVEETSRSEAMLKQMGLTKVFSRTEAQLGLLSEDPDVHVDEIVQFVN
dana            FQLKEMHVSLPKFQVEETSRSEAMLKQMGLKKLFSRTEAQLGLLSEDPDVHVDEIVQFVN
dpse            AQLRQMHVSLPKFQVEETSRSESMLKQMGLRRLFSRTEAQLGLLSEDPDVHVDEIVQFVN
dper            AQLRQMHVSLPKFQVEETSRSESMLKQMGLRRLFSRTEAQLGLLSEDPDVHVDEIVQFVN
dwil            SELKQMHVSLPKFQVEETSRSESVLKQLGLQRLFSRTDAELSLLSDDTDVHVDEIVQFVN
dmoj            ILLKELHVTLPKFQVMETSRSEAMLKQMGLKRLFSRTEAQLNLMSEDEDLHIDEIVQFVS
dvir            MQLKELHVTLPKFQVEETSRSEAMLQQLGLKRLFSRTEAQLGLLTEDEDVHVDEIVQFVN
dgri            LELRQLHVTLPKFQVEETSRSESMLKNLGLKRIFSRGEAQLGLLSEDEDLHIDEIVQFVN
                  ::::*:::***** ******::*:::** ::*** :*:*.*:::* *:*:*******.

dmel            VRVDEGGSSANSLSAATMQARTPSVESTVLPVPEPEPE-LP-GVERFEVNRPFAYFIVDC
dsim            VRVDEGGSSANSLSAATMQARTPSVESTVLPVPEPEPE-LP-GVERFEVNRPFAYFIVDC
dsec            VRVDEGGSSANSLSAATMQARTPSVESTVLPVPEPEPE-LP-GVERFEVNRPFAYFIVDC
dyak            VRVDEGGSSANSLSAATMQARTPSVESTVLPVPEPEPE-LP-GVERFEVNRPFAYFIVDC
dere            VRVDEGGSSANSLSAATMQARTPSVESTVLPVPEPEPE-LP-GVERFEVNRPFAYFIVDC
dana            VRVDEGGSSTNALSAANMQARTPSVESTVLPVPEPEP--EP-GVERFEVNRPFAYFLVDC
dpse            VRVDEGGSSANALSAATQARSPQAAEDSASVLPVPEPEPEP-GVERFEVNRPFAYFIMDC
dper            VRVDEGGSSANALSAATQARSPQAAEDSASVLPVPEPEPEP-GVERFEVNRPFAYFIMDC
dwil            VRVDEGGSSANALSAANMQARSPLASATELPVPEPEPE--P-GVERFEVNRPFAYFIMDC
dmoj            VRVDEDGSSTNSLSAGNIQARSPIAAEAAAVLPVPEPEPEP-GVERFDVNRPFAYFIVDC
dvir            VRVDEGGSSANSLSAATMQGRSPIAAEAAATLPVPEPEPEP-GVELFDVNRPFVYFVMDC
dgri            VRVDEGGSSANSLSAATTQARSPLFILQE---PEPEPEPEPEGVERFDVNRPFVYFVVDC
                *****.***:*:***..    .          * ***   * *** *:*****.**::**

dmel            QEQFVLASGKIYTPefkedlpsvsieveleqs
dsim            QEQFVLASGKIYTPefkedlpsvsveveleqs
dsec            QEQFVLASGKIYTPefkedlptvsveveleqs
dyak            QEQFVLASGKIYTPefkedlpsvsveveleqs
dere            QEQFVLASGKIYTpefkedlpsvsveveleqs
dpse            EEQFILASGKVYTpefkddlpgvsveieleqs
dper            EEQFVLASGKVYTpefkddlpgvsveieleqs
dana            EEQFVLASGKIYTpefkedlppvsieveleqs
dwil            ENQLVLASGKVYTpefkddlppvsveieleqs
dmoj            RTQVVLASGKVYApesvdelppvsieveleqs
dvir            KNQFVLASGKVYApefkdelppvsieveleqs
dgri            KNAFVLASGKIYApelkdelppvsieveleqs
                .  .:*****:*:**  ::** **:*:*****
```

Sequence stretches with serpin homology are in UPPER CASE. The PF residues at the C-terminal “shutter region” of RCL are in **RED** as are residues showing homology to the shutter-region consensus of inhibitory serpins.
